# Cost-effectiveness and Economic Benefit of Continuous Professional Development for Drug Prescribing: A Systematic Review

**DOI:** 10.1001/jamanetworkopen.2021.44973

**Published:** 2022-01-26

**Authors:** David A. Cook, Christopher R. Stephenson, John M. Wilkinson, Stephen Maloney, Jonathan Foo

**Affiliations:** 1 School of Continuous Professional Development, Mayo Clinic College of Medicine and Science, Rochester, Minnesota; 2 Division of General Internal Medicine, Mayo Clinic, Rochester, Minnesota; 3 Department of Family Medicine, Mayo Clinic, Rochester, Minnesota; 4 School of Primary and Allied Health Care, Monash University, Victoria, Australia

## Abstract

**Importance:**

The economic impact of continuous professional development (CPD) education is incompletely understood.

**Objective:**

To systematically identify and synthesize published research examining the costs associated with physician CPD for drug prescribing.

**Evidence Review:**

MEDLINE, Embase, PsycInfo, and the Cochrane Database were searched from inception to April 23, 2020, for comparative studies that evaluated the cost of CPD focused on drug prescribing. Two reviewers independently screened all articles for inclusion and reviewed all included articles to extract data on participants, educational interventions, study designs, and outcomes (costs and effectiveness). Results were synthesized for educational costs, health care costs, and cost-effectiveness.

**Findings:**

Of 3338 articles screened, 38 were included in this analysis. These studies included at least 15 659 health care professionals and 1 963 197 patients. Twelve studies reported on educational costs, ranging from $281 to $183 554 (median, $15 664). When economic outcomes were evaluated, 31 of 33 studies (94%) comparing CPD with no intervention found that CPD was associated with reduced health care costs (drug costs), ranging from $4731 to $6 912 000 (median, $79 373). Four studies found reduced drug costs for 1-on-1 outreach compared with other CPD approaches. Regarding cost-effectiveness, among 5 studies that compared CPD with no intervention, the incremental cost-effectiveness ratio for a 10% improvement in prescribing ranged from $15 390 to $437 027 to train all program participants. Four comparisons of alternative CPD approaches found that 1-on-1 educational outreach was more effective but more expensive than group education or mailed materials (incremental cost-effectiveness ratio, $18-$4105 per physician trained).

**Conclusions and Relevance:**

In this systematic review, CPD for drug prescribing was associated with reduced health care (drug) costs. The educational costs and cost-effectiveness of CPD varied widely. Several CPD instructional approaches (including educational outreach) were more effective but more costly than comparators.

## Key Points

**Question:**

What are the comparative costs and benefits of physician continuous professional development (CPD) for drug prescribing?

**Findings:**

In this systematic review of 38 studies, CPD was associated with reduced health care costs (median drug cost savings of $79 373) compared with no training. More intensive CPD (compared with no training or less intensive interventions) was associated with improved effectiveness (prescribing) outcomes but incurred greater education costs (incremental cost of $3 to $4105 per physician per standardized effectiveness change).

**Meaning:**

These results suggest that physician CPD for drug prescribing is associated with reduced health care costs and that both effectiveness outcomes and costs should be considered when making education decisions.

## Teaser

This systematic review of 38 studies assesses the association between the costs of physician continuous professional development and drug costs related to prescribing.

## Introduction

Inappropriate prescribing (including prescription errors, overuse and underuse of medications, and unnecessarily expensive medications) harms patients and wastes resources.^1^ Up to one-third of prescriptions are inappropriate,^2^ and these inappropriate prescriptions are associated with suboptimal clinical outcomes^3^ and large financial burden.^4,5^ With expanding drug formularies, increasing patient comorbidities, and progressively individualized treatment recommendations, ensuring appropriate prescribing will become even more challenging. Systematic reviews have found that restrictive interventions to optimize prescribing (eg, prioritized formularies, prior authorization, and computerized decision support) have benefits^6,7,8^ but may be associated with treatment delays and adverse effects on clinicians’ professional identity and culture.^6^ By contrast, educational interventions (eg, audit and feedback as well as educational outreach) have overall favorable effects on practitioner performance and patient outcomes.^6,9,10,11,12,13,14^

Continuous professional development (CPD) is essential to clinicians’ efforts to maintain competency after completion of training and includes formal educational interventions and unstructured learning activities. Although CPD programs to improve prescribing practices are increasingly common, their cost and cost-effectiveness remain incompletely characterized. Reviews^6,9,10,11,12,13,14^ of interventions to improve drug prescribing have touched only briefly on the economic outcomes (educational costs and health care cost savings) of CPD interventions. Only 1 review^15^ (published in 2002) focused on cost of CPD, and that review included only 1 study of CPD for drug prescribing.

A comprehensive synthesis of evidence regarding the comparative costs and benefits of physician CPD for drug prescribing would provide clinicians, educators, and administrators information to reduce wasted effort (cost and physician time), identify resource-efficient instructional approaches, and promote more effective health care. We conducted a systematic review to determine the comparative costs and benefits (including cost-effectiveness and health care cost savings) of physician CPD for drug prescribing and the CPD features that are associated with improved cost-benefit outcomes.

## Methods

This systematic review (part of a larger systematic review of economic outcomes of CPD^16^) was planned, conducted, and reported in adherence to the Preferred Reporting Items for Systematic Reviews and Meta-analyses (PRISMA) reporting guideline^17^ and reporting guidelines for economic evaluations.^18,19,20^

### Data Sources and Searches

We worked with an experienced reference librarian to create a search strategy to identify studies that examined the cost of CPD. This strategy incorporated key concepts and terms related to the population (eg, *physicians*), intervention (eg, *education continuing*), and outcomes^21^ (eg, *economics medical; costs and cost analysis*). We used this strategy (detailed in the eBox in the Supplement) to search MEDLINE, Embase, PsycInfo, and the Cochrane Database from each database’s inception through April 23, 2020. We subsequently identified 3 additional relevant studies cited in other included articles.

### Study Selection

We included all original comparative economic evaluation studies, published in any language, that investigated CPD for practicing physicians. We defined CPD as “activities intended to promote or measure the clinical knowledge/skills of physicians in independent medical practice through courses or assessments delivered in any modality or venue, whether or not continuing medical education (CME) credit is awarded; or self-directed learning or self-assessment activities for which CME credit is awarded.”^16^ (See the eBox in the Supplement for additional operational definitions.) From these studies we then identified all CPD activities that addressed the clinical topic of drug prescribing.

Reviewers (all authors) worked in assigned pairs to screen studies for inclusion, first reviewing the title and abstract and then reviewing the full text if needed (interreviewer agreement, κ = 0.73). Reviewers resolved all disagreements by consensus.

### Data Extraction and Quality Appraisal

We implemented a data abstraction form in software designed for systematic reviews (DistillerSR, Evidence Partners Inc). Two reviewers (D.A.C. and C.R.S.) independently extracted information on study design, participants, CPD interventions, methodological quality, effectiveness outcomes (including prescribing rates, errors, or costs), educational costs, and health care costs. For educational costs, we used Levin’s “ingredients” approach,^22^ organizing costs according to defined categories^16^ related to education planning and implementation. We resolved conflicts by consensus.

We appraised general methodological quality using the Medical Education Research Study Quality Instrument,^23^ which evaluates the study design, sampling, outcomes (type [counting drug prescriptions and drug costs as behavior outcomes], objectivity, and validity), and statistical analyses. We also appraised methods specific to economic analyses, selected from the 1996 *BMJ* guidelines.^24^

### Data Synthesis and Analysis

We converted all monetary estimates to 2021 US dollars, first adjusting for inflation in the original currency (starting with the final year of data collection and ending in 2021) and then converting the original currency into US dollars using exchange rates on May 6, 2021. Given well-documented concerns about meta-analysis of cost-effectiveness studies (including between-context heterogeneity in resource use and pricing^25,26,27,28^), we did not attempt such analyses. Instead, to evaluate cost-effectiveness, we adopted the dominance ranking matrix approach^29^ advocated by the Joanna Briggs Institute.^27^ We also calculated monetary net benefit (health care costs averted minus educational costs) for all studies that reported educational costs and health care costs.

## Results

We identified 3338 potentially eligible studies of which 38 met criteria for inclusion (eFigure 1 in the Supplement).^30,31,32,33,34,35,36,37,38,39,40,41,42,43,44,45,46,47,48,49,50,51,52,53,54,55,56,57,58,59,60,61,62,63,64,65,66,67^ These studies enrolled more than 15 659 health care professionals (median, 180.5; IQR, 68-353; 28 studies reporting this information) and collected data on more than 1 963 197 patients (median, 4763; IQR, 1622-169 447; 16 studies reporting). Three studies^40,42,62^ were translated from a non-English language (2 German and 1 Spanish). Five studies included more than 2 arms,^31,33,48,54,59^ resulting in more than 1 relevant comparison per study. After describing overall study features and quality, we report educational costs, health care cost impact, and educational costs combined with effectiveness outcomes (cost-effectiveness and net benefit).

### Key Features of Included Studies

Table 1 reports a summary of key features, and eTable 1 in the Supplement contains detailed information for each study. We distinguished studies that evaluated the cost of (expenses incurred in implementing) the CPD activity (12 studies^31,32,38,46,47,48,54,56,59,60,63,66^) and studies that evaluated health care costs after a CPD activity (35 studies^30,31,32,33,34,35,36,37,38,39,40,41,42,43,44,45,46,47,48,49,50,51,52,53,54,55,56,57,58,61,62,63,64,65,67^).

**Table 1.** Key Features of All Included Studies

Feature	Studies, No. (%) (N = 38)
Costs reported	
Educational costs	12 (32)
Health care (drug) costs	35 (92)
Participant specialty	
Family, internal, or general medicine	26 (68)
Internal medicine subspecialties	2 (5)
Surgery	2 (5)
Other specialties	3 (8)
Mixed unspecified specialties	11 (29)
Educational target	
Antibiotics	15 (39)
Analgesics	3 (8)
Antihyperlipidemics	3 (8)
Neuropsychiatric drugs	3 (8)
Gastrointestinal drugs	2 (5)
Antihistamines	2 (5)
Cardiovascular drugs	2 (5)
Radiology contrast dye	1 (3)
Multiple or nonspecific drugs	5 (13)
Prescribing errors	3 (8)
Instructional modality	
Paper materials	26 (68)
Small group	22 (58)
Audit and feedback	19 (50)
Large group	14 (37)
Case based	2 (5)
Internet	2 (5)
Video	2 (5)
Real or simulated patient	1 (3)
Noneducational clinical practice improvement	4 (11)
Study design	
≥2 Groups	
Randomized	12 (32)
Nonrandomized	12 (32)
1 Group pre/post or time series	14 (36)
Comparison	
No intervention (baseline or control group)	35 (92)
Alternate CPD or clinical intervention	10 (26)
>1 Investigator institution	7 (18)
≥6 Participant sites	25 (66)
Original currency	
US dollar	18 (47)
British pound	5 (13)
Euro	5 (13)
Canadian dollar	3 (8)
Other^a^	6 (16)
Not reported	1 (3)
Validity evidence	
Content	10 (26)
Internal structure	5 (13)
High participant retention (≥75%)	27 (71)
Overall high quality (MERSQI score ≥12)	16 (42)

Abbreviations: CPD, continuous professional development; MERSQI, Medical Education Research Study Quality Instrument.

^a^ Other currencies include German deutschmark, Danish kroner, Swiss franc, Australian dollar, and Saudi riyal.

All studies involved physicians in independent practice, most of whom were family, internal, or general medicine physicians (26 studies [68%]) or all physicians in the facility (4 studies [11%]). Thirteen studies^33,35,41,47,49,51,52,54,55,56,57,61,66^ (34%) involved other health care professionals (besides physicians), including postgraduate physician trainees (n = 5), nurse practitioners and physician assistants (n = 5), pharmacists (n = 5), nurses (n = 3), and medical students (n = 1).

Most studies focused on optimal use of specific drugs or drug classes, including antibiotics (n = 15 studies), analgesics (n = 3 studies), antihyperlipidemics (n = 3 studies), and neuropsychiatric drugs (n = 3 studies). Three studies focused on general drug-drug interactions or prescribing errors. One study^66^ compared 2 approaches to increase attendance at a CPD course on drug prescribing.

Instructional modalities included paper materials (26 studies^30,31,32,33,34,35,36,37,38,39,41,45,47,48,49,50,51,52,53,54,55,60,61,63,64,67^), face-to-face small groups or 1-on-1 outreach (22 studies^30,31,33,35,36,40,42,43,44,46,48,49,50,53,54,56,58,59,60,62,63,65^), performance audit and feedback (19 studies^35,37,38,39,42,43,44,45,46,49,51,52,53,57,58,59,63,64,65^), and face-to-face large groups (14 studies^30,32,34,35,37,43,47,52,55,56,57,59,60,65^). Four studies^41,51,57,67^ also implemented changes in clinical practice (eg, clinical checklists).

Considering the context of education, most studies were conducted in private or independent practice^31,32,33,34,35,36,37,38,40,41,42,43,44,45,46,47,48,49,50,53,54,55,56,58,59,60,61,63,64,65,66,67^ (32 [84%]); 6 (16%) were in academic settings^30,34,39,40,52,57^ and 2 (5%) in government facilities.^51,62^ Thirty studies^31,33,34,35,38,40,41,42,43,44,45,46,47,48,49,50,51,53,54,55,56,58,59,60,61,63,64,65,66,67^ (79%) involved patients in an ambulatory setting, 7 (18%) involved patients in a hospital ward,^30,32,36,37,52,57,62^ and 3 (8%) involved a medical procedure.^32,37,39^ Geographic origins included the US (15 [39%]), the UK (5 [13%]), other European countries (11 [29%]), Asia (3 [8%]), Canada (3 [8%]), and Oceania (1 [3%]). Funding sources included industry^30,42,49,58,61^ (5 [13%]), government^31,32,48,50,51,59,63,64,65,66^ (10 [26%]), private^34,53^ (2 [5%]), and local^57^ (1 [3%]) support. Twenty studies (53%) did not report funding^33,35,36,37,38,39,40,41,43,44,45,46,47,52,54,55,56,60,62,67^; among these, for 5 studies^33,35,41,43,44^ the authors were employed by a capitated health plan (ie, a potential conflict of interest).

### Study Quality

Twelve studies (32%) used randomized group assignment,^31,33,45,48,50,53,54,55,59,63,64,65^ 12 (32%) used 2 or more nonrandomized groups,^32,35,38,41,44,46,47,58,60,61,66,67^ and 14 (36%) involved a single-group pre/post or time-series design.^30,34,36,37,39,40,42,43,49,51,52,56,57,62^ Nearly all studies (35 [92%]) compared CPD against no intervention^30,31,32,33,34,35,36,37,38,39,40,41,42,43,44,45,46,47,48,49,50,51,52,53,56,57,58,59,60,61,62,63,64,65,67^ (baseline performance or a no intervention control group), whereas 10 (26%) compared one CPD intervention against another^31,33,48,50,54,55,59,60,65,66^ (eg, comparing different instructional approaches or implementation strategies).

Outcome measures were blinded or obtained from an unbiased data source (eg, pharmacy claims database) in 22 studies^31,38,39,41,43,44,45,46,47,48,50,51,53,54,55,56,58,61,63,64,65,67^ (58%). Evidence to support the validity of the outcome measure was infrequently reported: 10 studies (26%) reported content evidence^32,45,46,48,51,57,59,63,64,67^ (eg, information about the database) and 5 (13%) reported internal structure evidence^33,41,48,51,64^ (such as interrater reliability); none reported correlation with another variable. Participant retention was high in 27 studies^31,32,33,38,39,40,41,42,43,44,45,46,47,48,50,51,53,54,55,56,58,59,61,63,64,65,67^ (71%) (explicitly reported as ≥75% or data obtained from a database presumed to reflect all eligible clinicians). Thirty-two studies^30,31,32,33,34,35,37,38,39,40,41,42,43,45,46,47,48,50,53,54,55,56,57,58,59,61,62,63,64,65,66,67^ (84%) specified the time horizon, 3 (8%) stated a discount rate^24^ or justified its absence,^31,33,63^ and 3 (8%) conducted a sensitivity analysis.^31,48,64^ See eTable 2 in the Supplement for additional details of economic study quality. Methodological quality (Medical Education Research Study Quality Instrument) scores ranged from 7 to 16 (of 18 possible), with a mean (SD) of 11.4 (2.3) (median, 11.5; IQR, 9.5-13.0).

### Educational Costs

Twelve studies^31,32,38,46,47,48,54,56,59,60,63,66^ (32%) reported costs associated with implementing CPD interventions. For each study, we extracted 12 possible cost ingredients^16,22^ (Table 2). The ingredients reported varied widely across studies. Three studies (5 interventions) reported only the total estimated educational cost without any mention of specific ingredients, and another 3 studies (4 interventions) reported some or all costs as a lump sum while delineating the ingredients that counted in that sum. The most commonly reported ingredient was the cost of course faculty (7 studies), whereas 6 counted postage and 5 included the cost of educational materials. Four studies reported specific costs for 5 ingredients or more, and 6 studies reported costs for 3 ingredients or more. No studies itemized costs for equipment. Data sources for educational costs were reported infrequently, with 3 studies^59,60,63^ (25%) reporting details of ingredient quantification and 5 studies^38,56,59,60,63^ (42%) reporting details of pricing.

**Table 2.** Educational Costs of Physician Continuous Professional Development

Source and intervention	Cost, $^a^														No. trained
	Attendee time	Facility	Administrator time	Faculty time	Postage	Information/ communication technologies	Materials	Overhead	Travel	Catering	Publicity	Merged^b^	Unspecified	Total cost	
Soumerai et al,^31^ 1986															
Outreach	NA	NA	5970	16 002	0	0	4681	5563	5021	NA	0	1313	NA	38 550	141
Mailed	NA	NA	2151	384	NA	NA	3227	307	NA	NA	NA	NA	NA	6069	132
Landgren et al,^32^ 1988, multimodal	NA	NA	NA	NA	NA	NA	NA	NA	NA	NA	NA	NA	153 739	153 739	NA
Zimmerman et al,^38^ 1994, audit and feedback	NA	NA	NA	NA	NA	NA	NA	NA	NA	NA	NA	NA	20 783	20 783	NA
McNulty et al,^46^ 2000, workshop	NA	NA	NA	NA	NA	NA	NA	NA	NA	1847	NA	NA	NA	1847	101
Valori et al,^47^ 2001, workshop and mailed	NA	NA	NA	14 582	754	NA	1257	NA	NA	NA	NA	NA	NA	16 593	123
Watson et al,^48^ 2001															
Outreach and mailed	0	NA	NA	0	0	NA	0	NA	0	NA	NA	6028	NA	6028	35
Mailed	NA	NA	NA	NA	0	NA	0	NA	NA	NA	NA	281	NA	281	36
Simon et al,^54^ 2005															
Outreach (1-on-1)	NA	NA	NA	NA	NA	NA	NA	NA	NA	NA	NA	NA	8450	8450	114
Mailed	NA	NA	NA	NA	NA	NA	NA	NA	NA	NA	NA	NA	1690	1690	133
Outreach (group)	NA	NA	NA	NA	NA	NA	NA	NA	NA	NA	NA	NA	5915	5915	120
Siriwardena et al,^56^ 2007, lecture and discussion	15 856	28 288	44 954	4355	NA	NA	NA	NA	NA	NA	NA	NA	NA	93 453	68
Lopez-Picazo et al,^59^ 2011															
Outreach (1-on-1)	NA	NA	NA	15 200	818	2723	NA	NA	371	750	NA	NA	NA	19 862	69
Outreach (group)	NA	NA	NA	11 061	830	2723	NA	NA	273	761	NA	NA	NA	15 648	70
Mailed	NA	NA	NA	NA	794	2723	NA	NA	NA	728	NA	NA	NA	4245	67
Qureshi et al,^60^ 2011															
Multimodal	24 474	NA	NA	11 173	NA	NA	NA	2149	NA	NA	NA	NA	NA	37 796	61
Mailed	NA	NA	NA	6448	11 177	NA	1719	860	NA	NA	NA	NA	NA	20 204	520
Butler et al,^63^ 2012, multimodal	136 362	NA	13 046	31 398	0	NA	826	NA	1922	NA	NA	NA	NA	183 554	127
Holuby et al,^66^ 2015															
Formal marketing	NA	NA	0	NA	NA	NA	NA	NA	NA	NA	0	15 679	NA	15 679	31
Informal publicity	NA	NA	3126	NA	NA	NA	NA	NA	NA	NA	NA	NA	NA	3126	48
Mean, $^c^	58 897	28 288	13 849	12 289	2875	2723	2342	2220	1897	1022	NA	NA	NA	32 676	NA
Frequency, No.^d^	4	1	4	7	6	2	5	2	4	2	2	3	3	20	-

Abbreviation: NA, not available (ingredient not mentioned in the study).

^a^ All costs have been converted to 2021 US dollars. Zero indicates ingredient listed as part of a merged cost but not separately quantified. We also sought but found no studies that reported costs for equipment.

^b^ Merged reflects costs for which the constituent ingredients (indicated by 0 in the corresponding column) were listed but not separately quantified.

^c^ Mean excludes 0 cells (ie, those counted as part of a merged quantity).

^d^ Frequency indicates number of studies reporting this ingredient (counted only once per study) and includes studies with 0 cells.

The mean ingredient cost per intervention varied widely, ranging from $58 897 for learner attendance (ie, compensated hourly or daily wage or estimated lost revenue; mean of 3 interventions) (all costs reported in 2021 US dollars) to $1022 for catering (mean of 2 interventions). The total reported educational cost likewise varied widely, ranging from $281 (a study^45^ that analyzed only postage and paper and reached 36 physicians) to $183 554 (a study^63^ that analyzed 6 ingredients, training 127 physicians), with a mean (SD) of $32 676 ($51 215) (median, $15 664; IQR, $5080-$29 290).

### Economic Outcomes (Health Care Cost Impact)

Thirty-five studies^30,31,32,33,34,35,36,37,38,39,40,41,42,43,44,45,46,47,48,49,50,51,52,53,54,55,56,57,58,61,62,63,64,65,67^ (92%) evaluated health care costs after a CPD activity (see Table 3 for key features). In 11 studies^32,34,49,52,53,55,57,61,62,63,65^ the CPD intervention focused on wise stewardship (using the most appropriate drug [eg, antibiotic] for a given condition), 10 studies^36,37,39,41,43,44,45,50,51,67^ encouraged choice of less expensive drugs, 7 studies^30,38,40,42,46,47,58^ targeted fewer prescriptions overall, and 7 studies^31,33,35,48,54,56,64^ encouraged increased use of an effective treatment.

**Table 3.** Key Features of Studies That Measured Health Care Cost Impact (Drug Cost Savings)

Feature	Studies, No. (%) (N = 35)
Comparison	
No intervention (baseline or control group)	33 (94)
Alternate CPD or clinical intervention	7 (20)
Intent of education	
Wise stewardship	11 (31)
Less expensive drug choice	10 (29)
Increase effective treatment	7 (20)
Fewer prescriptions	7 (20)
Comparison cohort appropriate	
Concurrent	10 (29)
Matched for season	15 (43)
Blinding of outcome	22 (63)
Quantification of drug use	
Claims database	19 (54)
Medical record review	3 (9)
Physician self-report	2 (6)
Undefined	11 (31)
Pricing of drug	
Average wholesale price	5 (14)
Actual (claims database)	18 (51)
Government publication	1 (3)
Other	2 (6)
Undefined	9 (26)
Duration of data collection after CPD, mo	
≤1	4 (11)
1-11	15 (43)
≥12	11 (31)
Not reported	5 (14)
Drug appropriateness	
Confirmed	5 (14)
Extrapolation of findings	
Different sample size	25 (71)
Different duration	9 (26)

Abbreviation: CPD, continuous professional development.

All 35 studies^30,31,32,33,34,35,36,37,38,39,40,41,42,43,44,45,46,47,48,49,50,51,52,53,54,55,56,57,58,61,62,63,64,65,67^ reported an outcome of drug cost, and this outcome was often the only health care cost outcome; thus, we used drug cost as the (common) metric of health care costs. For 1 study^56^ the investigators expected higher short-term drug costs if prescribing recommendations were followed (ramipril in patients with diabetes); for the other 34 studies,^30,31,32,33,34,35,36,37,38,39,40,41,42,43,44,45,46,47,48,49,50,51,52,53,54,55,57,58,61,62,63,64,65,67^ the economic goal appeared to be immediate cost savings. The appropriateness of drug prescription was appraised in 5 studies^32,33,37,57,67^ (14%). Twenty-five studies^33,38,46,47,54,55,58,63,64,65^ took steps to ensure an appropriate (less biased) comparison by using a concurrent control group^33,38,46,47,54,55,58,63,64,65^ (10 [29%]) or making historical comparison matched for time of year^30,31,32,34,40,41,42,45,46,55,56,58,63,64,65^ (15 [43%]). Twenty-four studies^31,32,33,34,37,38,39,41,43,46,48,50,51,52,53,54,55,56,58,61,63,64,65,67^ (69%) reported details of resource quantification, and 26 studies^31,32,33,34,35,38,39,41,43,44,45,46,47,48,50,51,52,54,56,57,58,61,63,64,65,67^ (74%) reported details of pricing. Thirty studies^30,31,32,33,34,35,37,38,39,40,41,42,43,45,46,47,48,50,53,54,55,56,57,58,61,62,63,64,65,67^ (86%) reported the period of data collection, which ranged from 1 to 182 weeks.

#### Comparisons of CPD With No Intervention

Thirty-three studies (37 comparisons) evaluated drug costs for CPD compared with no intervention (a separate control group^31,32,33,35,38,41,43,44,45,46,47,48,50,53,58,61,63,64,65,67^ [n = 23 comparisons] or baseline metrics^30,34,36,37,39,40,42,43,49,51,52,56,57,62^ [n = 14 comparisons]). In all but 2 instances,^56,65^ these studies found that CPD was associated with lower drug costs (Table 4). Considering these exceptions, in 1 study^56^ CPD led to increased prescribing of ramipril as intended for patients with diabetes and a concomitant (expected) increase in drug costs. In the other study,^65^ the rate and cost of antibiotic prescriptions were reduced as intended, but recommendations for nonantibiotic symptomatic treatments increased, resulting in higher total drug costs.

**Table 4.** Health Care Cost Impact (Drug Cost Savings)^a^

Source and intervention	Total savings (period in wk), $^b^	No. of physicians/No. of patients^c^	Savings, %^d^	Savings per 100 patients per y, $^e^	Savings per 100 physicians per y, $^e^
**Comparison with alternate CPD approach**					
Soumerai et al,^31^ 1986, outreach vs mailed	27 491 (39)	273/NR	8.5	NA	19 497
Raisch et al,^33^ 1990, outreach: case studies vs statistical information	NA	NR/NR	−26.8	−9865	NA
Watson et al,^48^ 2001, outreach (1-on-1) and mailed vs mailed	NA	NR/NR	9.2	4	NA
Bernal-Delgado et al,^50^ 2002, outreach vs conventional	NA	102/NR	1.8	NA	NA
Simon et al,^54^ 2005					
Outreach (1-on-1) vs mailed	NA	NR/NR	3.6	1690	NA
Outreach: 1-on-1 vs group	NA	NR/NR	10.8	5070	NA
Mailed vs outreach (group)	NA	NR/NR	6.9	3380	NA
Chazan et al,^55^ 2007, seminar: continuous vs seasonal	18 978 (13)	400/168 644	77.0	83	NA
Le Corvoisier et al,^65^ 2013, seminar: role play and reflection vs less interactive	NA	72/NR	NA	NA	395 086
**Comparison with no intervention**					
Moleski et al,^30^ 1986, post-pre (multimodal)	319 800 (52)	NR/NR	62.0	NA	NA
Soumerai et al,^31^ 1986					
CPD vs NI (outreach)	43 083 (39)	281/NR	13.0	NA	40 740
CPD vs NI (mailed)	14 597 (39)	272/NR	4.5	NA	11 058
Landgren et al,^32^ 1988, CPD vs NI (multimodal)	267 243 (104)	NR/NR	24.0	NA	NA
Raisch et al,^33^ 1990					
CPD vs NI (outreach with statistics)	NA	NR/NR	46.1	31 476	NA
CPD vs NI (outreach with case studies)	NA	NR/NR	31.7	21 612	NA
Friis et al,^34^ 1991, post-pre (multimodal)	3126 600 (52)	NR/NR	NA	NA	NA
Stuart et al,^35^ 1991, CPD vs NI (multimodal)	NA	NR/NR	51.0	331	NA
Hadbavny et al,^36^ 1993, post-pre (multimodal)	90 792 (52)	NR/1095	NA	NA	NA
Weir,^37^ 1993, post-pre (multimodal)	9433 (52)	27/550	24.0	1714	NA
Zimmerman et al,^38^ 1994, CPD vs NI (audit and feedback)	61 280 (52)	NR/2139	26.5	15 359	NA
Ziskind et al,^39^ 1994, post-pre (audit and feedback)	403 962 (52)	5/2974	60.9	26 169	NA
Bausch,^40^ 1995, post-pre (discussion group)	1153 129 (104)	11/NR	9.3	NA	5 241 495
Schectman et al,^41^ 1996, CPD vs NI (education and clinical)	26 901 (52)	33/35 000	4.4	77	NA
von Ferber et al,^42^ 1997, post-pre (discussion group)	NA	NR/NR	NA	186 862	NA
Boreen et al,^43^ 1998					
Post-pre (outreach)	67 953 (52)	335/NR	2.8	NA	20 285
CPD vs NI (outreach)	NA	NR/NR	3.7	7848	NA
Hux et al,^45^ 1999, CPD vs NI (audit and feedback)	NA	NR/NR	31.7	5223	NA
Unnamed,^44^ 1999, CPD vs NI (audit and feedback with outreach)	NA	NR/NR	34.8	78 368	NA
McNulty et al,^46^ 2000, CPD vs NI (workshop)	31 455 (17)	339/NR	5.1	63 925	NA
Valori et al,^47^ 2001, CPD vs NI (workshop and mailed)	216 526 (26)	123/325 000	14.7	133	NA
Watson et al,^48^ 2001					
CPD vs NI (outreach and mailed)	NA	NR/NR	12.2	5	NA
CPD vs NI (mailed)	NA	NR/NR	3.0	1	NA
Bell,^49^ 2002, post-pre (multimodal)	NA	NR/NR	17.8	7441	NA
Bernal-Delgado et al,^50^ 2002, CPD vs NI (outreach)	NA	104/NR	3.7	NA	NA
Dobscha et al,^51^ 2003, post-pre (multimodal)	46 449 (4)	NR/2909	22.6	16 646	NA
Lutters et al,^52^ 2004, post-pre (multimodal)	4731 (1)	NR/284	54.0	86 862	NA
Madridejos-Mora et al,^53^ 2004, CPD vs NI (multimodal)	464 391 (17)	282/500 000	22.7	4100	NA
Siriwardena et al,^56^ 2007, post-pre (multimodal)	−637 615 (52)	NR/NR	NA	NA	NA
Apisarnthanarak et al,^57^ 2010, post-pre (multimodal)	38 886 (78)	NR/412	NA	6293	NA
Niquille et al,^58^ 2010, CPD vs NI (discussion group)	6 912 000 (52)	24/NR	42.0	NA	28 800 000
Weiss et al,^61^ 2011, CPD vs NI (mailed)	NA	NR/NR	12.5	239	NA
del Arco et al,^62^ 2011, post-pre (outreach)	46 782 (52)	NR/281	NA	16 648	NA
Butler et al,^63^ 2012, CPD vs NI (multimodal)	NA	NR/NR	5.5	23	NA
Dormuth et al,^64^ 2012, CPD vs NI (audit and feedback + mailed)	147 842 (52)	NR/19 049	6.0	3576	NA
Le Corvoisier et al,^65^ 2013, CPD vs NI (multimodal)	NA	171/NR	NA	NA	−87 527
Pechlivanoglou et al,^67^ 2015, CPD vs NI (multimodal)	3 415 392 (182)	NR/170 550	NA	NA	NA

Abbreviations: CPD, continuous professional development; NA, not available (insufficient information to calculate); NI, no intervention; NR, not reported.

^a^ All costs and monetary benefits have been converted to 2021 US dollars. Post-pre indicates 1-group pretest-posttest comparison.

^b^ Total health care (drug) costs averted during the study or as extrapolated by the study authors and the time period (actual or extrapolated) encompassed in this analysis.

^c^ Number of physicians and patients encompassed in the total savings analysis.

^d^ Savings compared with baseline or comparison group (positive numbers favor the first intervention listed in comparison).

^e^ Savings adjusted to reflect 100 patients during 12 months (eg, 1200 1-month supplies or 36 500 daily doses) or 100 procedures (eg, 100 courses of postsurgical antibiotic prophylaxis) or 100 physicians during 12 months.

Among the remaining 31 studies,^30,31,32,33,34,35,36,37,38,39,40,41,42,43,44,45,46,47,48,49,50,51,52,53,57,58,61,62,63,64,67^ the total reported savings ranged from $4731 to $6 912 000 (median, $79 373; n = 22 studies reporting information sufficient to calculate), and the percentage of savings compared with the control group or baseline costs ranged from 2.8% to 62.0% (n = 29 studies). Substantial variation in study time period and sample size precluded meaningful cross-study comparisons of total costs, so when possible we standardized these variables to reflect a fixed period and number of patients or physicians (100 for 1 year). Although admittedly imperfect (it implies a linear response across time and sample sizes, and cross-study cost comparisons are still inherently challenging), this approach offers useful insights. Savings for 100 patients ranged from $1.00 to $186 862 per year (n = 24 studies), and savings for 100 physicians ranged $11 058 to $28 800 000 per year (n = 5 studies).

#### Comparisons of Alternative CPD Approaches

Seven studies^31,33,48,50,54,55,65^ (9 comparisons) compared drug costs for 2 or more alternative CPD approaches (Table 4). Four studies (5 comparisons) found that 1-on-1 outreach education was associated with reduced drug costs in comparison with mailed materials,^31,48,54^ group education,^54^ or conventional CPD.^50^ Studies also found reduced drug costs for seminars with added role play and self-reflection activities (vs less interactive seminars)^65^ and year-round (vs seasonal) activities.^55^ Contrary to their authors’ hypotheses, studies found higher drug costs for group outreach (vs mailed materials)^54^ and for 1-on-1 outreach augmented with vivid case studies (vs augmented with statistical information from a review article).^33^

### Cost-effectiveness and Net Benefit

#### Comparisons of CPD With No Intervention

Nine studies^31,32,38,46,48,56,59,60,63^ (24%) reported costs and clinical (prescribing) outcomes for CPD compared with no education (a no intervention control group or baseline metrics). Eight of these studies^32,38,46,48,56,59,60,63^ reported an effectiveness outcome (rate of drug prescribing [n = 5], prescribing errors [potential drug-drug interactions or errors in writing; n = 2], prescription counts [n = 1]), thus allowing estimation of the incremental cost-effectiveness ratio (ICER). All these studies^32,38,46,48,56,59,60,63^ found improved prescribing outcomes with CPD, but costs were of course higher than for no education (Table 5). The dominance ranking matrix (eFigure 2 in the Supplement) classifies such studies as unclear dominance, and decisions require judgment of “whether intervention [is] preferable considering incremental cost effectiveness measures and priorities/willingness to pay.”^27^

**Table 5.** Cost-effectiveness and Net Benefit

Source and comparison	Effectiveness outcome^c^	Incremental effectiveness, unadjusted^d^	Incremental cost, $^a^				Net benefit, $^e^
			Total incremental cost	Cost per standardized change^b^			
				Total cost (% change)	Per physician (No. of physicians)	Per patient (No. of patients)	
**Comparison with alternate CPD approach**							
Lopez-Picazo et al,^59^ 2011, outreach: 1-on-1 vs group	Prescription errors	3.3% Fewer (errors)	4216	1263 (1)	18 (69)	0.03 (42 147)	NA
Qureshi et al,^60^ 2011, multimodal vs mailed	Prescription errors	1.3% Fewer (errors)	20 204	13 533 (1)	3 (5261)	68 (200)	NA
Soumerai et al,^31^ 1986, outreach vs mailed	None (prescription cost)^f^	NA	NA	NA	NA	NA	221 per physician
Watson et al,^48^ 2001, outreach (1-on-1) and mailed vs mailed	Prescription rate	0.4% Fewer	5746	143 664 (10)	4105 (35)	NA	NA
Simon et al,^54^ 2005, outreach: 1-on-1 vs group	Prescription rate (more)	3.4% More (preferred)	2535	7456 (10)	65 (114)	3.73 (2000)	48.00 per patient
Simon et al,^54^ 2005, outreach (1-on-1) vs mailed	Prescription rate (more)	4.6% More (preferred)	6760	14 696 (10)	129 (114)	7.35 (2000)	10.00 per patient
**Comparison with no intervention**							
Lopez-Picazo et al,^59^ 2011, CPD vs NI (outreach 1-on-1)	Prescription errors	15.4% Fewer (errors)	19 862	1294 (1)	19 (69)	0.03 (38 309)	NA
Qureshi et al,^60^ 2011, post-pre (multimodal)	Prescription errors	11.6% Fewer (errors)	37 796	3258 (1)	53 (61)	2.54 (1282)	NA
Soumerai et al,^31^ 1986, CPD vs NI (outreach)	None (prescription cost)^f^	NA	NA	NA	NA	NA	323 per physician
Siriwardena et al,^56^ 2007, post-pre (multimodal)	Prescription count (more)	52 345 New prescriptions	93 453	179 (100)	NA	NA	NA
Zimmerman et al,^38^ 1994, CPD vs NI (audit and feedback)	Prescription rate	8.9% Fewer	20 783	23 351 (10)	NA	11 (2139)	19.00 per patient
McNulty et al,^46^ 2000, CPD vs NI (workshop)	Prescription rate	1.2% Fewer	1847	15 390 (10)	152 (101)	NA	NA
Watson et al,^48^ 2001, CPD vs NI (outreach and mailed)	Prescription rate	2.1% Fewer	6029	28 707 (10)	820 (35)	NA	NA
Butler et al,^63^ 2012, CPD vs NI (multimodal)	Prescription rate	4.2% Fewer	183 555	437 027 (10)	3441 (127)	0.91 (479 502)	−879 per physician
Landgren et al,^32^ 1988, CPD vs NI (multimodal)	Prescription rate (more)	11.3% More (appropriate)	153 739	136 053 (10)	NA	21 (6415)	4.00 per patient^g^

Abbreviations: CPD, continuous professional development; NA, not available (insufficient information to calculate); NI, no intervention.

^a^ All costs and monetary benefits have been converted to 2021 US dollars.

^b^ Standardized change indicates 10% change in prescribing rate, 1% change in prescription error (potential drug-drug interaction or error in writing or wording of the prescription), or 100 prescriptions. The standardized change is intended only for relative comparisons across studies and should not be interpreted literally; the adjustment implies that increased (or decreased) monetary investment would lead to a linear improvement (or decline) in outcomes, which is unlikely to be true.

^c^ Effectiveness outcome indicates that the intended change (explicit or implied) was fewer for each outcome, unless otherwise noted as more.

^d^ Incremental effect of first intervention compared with second.

^e^ Net benefit was calculated as total health care (drug) costs averted minus total cost of education per trained physician or per patient. Positive values indicate favorable balance (ie, health care savings greater than educational costs). Costs reflect 12 months unless otherwise noted.

^f^ Reported the cost but not the quantity of drugs prescribed.

^g^ Drug costs averted extrapolated to 2 years.

To permit cross-study comparisons, we standardized the ICERs using change thresholds that reflect a subjectively meaningful difference, namely a 10% change in prescribing rates, a 1% change in prescribing errors, or an absolute change of 100 prescriptions. The standardized ICER for a 10% improvement in prescribing rates ranged from $15 390 to $437 027 to train all program participants (5 studies). When further standardized to 1 physician trained, the ICERs ranged from $152 to $3441 per physician. Standardized ICERs for other outcomes ranged from $179 to $3258 per physician.

Four studies^31,32,38,63^ (11%) reported information that allowed estimation of the net benefit (ie, health care cost savings minus educational costs) for CPD compared with no education. Three studies^31,32,38^ found a favorable net benefit (Table 5). The fourth study^63^ found that educational costs exceeded health care savings (net benefit of −$879 per physician trained), but extrapolation of benefits suggested a break-even point (ie, when savings [costs averted] equal educational costs) approximately 3.5 years after the intervention.

#### Comparisons of Alternative CPD Approaches

Studies that compare the cost-effectiveness of alternative educational approaches are vital to efforts to optimize CPD. Five studies^31,48,54,59,60^ (13%) (6 comparisons) compared the costs and clinical (prescribing) outcomes of 2 or more alternative CPD approaches (Table 5). All these studies^31,48,54,59,60^ found improved prescribing after enhancements intended to improve educational effectiveness but at the expense of increased education costs (ie, unclear dominance) (eFigure 2 in the Supplement). Specifically, 3 studies^48,54,59^ (4 comparisons) found that 1-on-1 educational outreach was more effective but more expensive than group education or mailed materials (ICERs ranging from $18 to $4105 per physician trained). Two other studies^31,60^ compared face-to-face instruction with mailed materials; one study^60^ found that face-to-face instruction was more effective but more expensive (ICER of $3 per physician trained), and the other study^31^ found a net benefit of $221 per physician favoring face-to-face instruction. One additional study^66^ compared 2 approaches to recruit participants to a CPD activity on drug prescribing and found that informal publicity through local organizations was more effective (higher attendance) and less expensive than a formal advertising campaign (ie, the informal approach dominated the formal).

## Discussion

This systematic review examined the costs and economic benefits of CPD for practicing physicians. Among studies^30,31,32,33,34,35,36,37,38,39,40,41,42,43,44,45,46,47,48,49,50,51,52,53,54,55,56,57,58,61,62,63,64,65,67^ that evaluated health care costs (drug costs), we found that CPD is associated with substantial cost savings. Among studies^31,32,38,46,47,48,54,56,59,60,63,66^ that evaluated educational costs, cost-effectiveness estimates varied widely, even after attempts to standardize the outcome measures. Along with expected differences in local contexts, educational approaches, and effectiveness outcomes, this heterogeneity arises from large differences in the accounting of educational costs. For example, only 4 studies accounted for 5 cost ingredients or more in their cost estimates, and 3 studies reported no discrete ingredients. Across all studies, we found that different CPD approaches can be associated with substantial differences in health care costs, educational costs, and incremental cost-effectiveness.

Studies reported information on participants (physicians and patients) inconsistently. Studies likewise frequently omitted details on ingredient (resource) quantification and pricing for educational costs (missing in >50%) and drug costs (missing in >25% of studies).

Previous reviews^6,9,10,11,12,13,14^ of drug prescribing have suggested that outreach, audit and feedback, and multimodal educational interventions are consistently associated with improved drug prescribing (ie, effectiveness). Our review extends these findings by examining cost along with effectiveness and confirms that 1-on-1 educational outreach is effective but incurs higher cost. Audit and feedback was frequently used in the studies we identified, but study designs did not allow direct cost comparisons.

The findings of this review support 4 important messages. First, CPD was associated with substantially reduced drug costs (in 31 of 33 studies^30,31,32,33,34,35,36,37,38,39,40,41,42,43,44,45,46,47,48,49,50,51,52,53,57,58,61,62,63,64,67^; median immediate savings of $79 373). Two studies^55,65^ found increased costs, but in 1 study^56^ this was intentional (increased prescribing of a drug with long-term health benefits), and in the other^65^ there was a decrease in the prescription rate and cost of the target drug (antibiotics) despite an increase in overall drug costs. These exceptions underscore the importance of explicitly stating and justifying the intended impact (eg, fewer prescriptions, more prescriptions, wise stewardship, or cheaper drug choices) and illustrate that focused, immediate outcomes may differ from broad, delayed economic measures.

Second, our findings highlight differences in effectiveness and cost among different CPD approaches. Most notably, 1-on-1 educational outreach was more effective but more costly than comparators. Indeed, all cost-effectiveness studies^,^^32,38,46,48,54,56,59,60,63^ had unclear dominance (and the Dominance Ranking Matrix^27,29^ [eFigure 2 in the Supplement] was unfortunately not very helpful in this synthesis). In the absence of dominance, local factors, resources, priorities, and values will influence local decisions. These findings underscore the importance of moving beyond questions of effectiveness alone (“What works?”) and adding economic data to inform judgments of value (“Is it worth the investment?”).^68,69,70^

Third, our findings indicate that some ingredients are more important (ie, contribute more to total cost) than others. We observed a 50-fold difference in highest vs lowest mean cost per ingredient. The most expensive ingredients reflected time (opportunity cost of lost wages or productivity) invested by learners, administrators, and faculty, and facility costs. Indeed, itemized time expenses collectively represented 55% of all educational costs (Table 2), and the total would likely have been greater if all studies had itemized time. Acknowledging that these data are far from definitive and that specifics will vary for other interventions and topics, our findings offer guidance in prioritizing cost ingredients. We urge that time—both quantity and price—for learners, teachers, and administrators be measured and reported accurately and consistently in economic studies of education.

Fourth and most importantly, our findings amplify prior requests for more and better educational cost evaluations.^22,71,72^ Our method to standardize educational costs, cost-effectiveness, and net benefits to a common time period, sample size, and level of effectiveness offers an example that others may choose to adopt when synthesizing economic data. However, this synthesis would have been facilitated by more complete accounting of educational costs,^22^ more consistent measurement of clinical outcomes (effectiveness and health care costs using standardized metrics and defined periods of follow-up), and better reporting^16,18,24,71^ in the original studies. We encourage education researchers to measure and report costs more frequently, robustly, and completely.

### Strengths and Limitations

This study has several strengths, including the robust literature search, duplicate review at all stages, and synthesis of key study findings using recommended approaches^27^ and a variety of tabular summaries.

This review also has some limitations. This study was limited by poor reporting in many original studies, which impaired our ability to identify key elements of methods and results. In particular, data on the number of physicians trained or patients treated were reported inconsistently, such that it was not always possible to rescale data to a common unit that allowed straightforward synthesis or comparison across studies. In addition, both effectiveness and costs are sensitive to numerous design features, including sample size, outcome selection and measurement, and duration of follow-up (all of which varied widely). Robust standardization would require a direct association among cost, effectiveness, and scale (eg, sites, participants, and duration), which is unlikely.^68^ Moreover, differences in local systems, funding, culture, and values will constrain local applications of cost-related outcomes regardless of rigor in the original data collection or meta-synthesis.^25,27,28,68^ However, we propose that syntheses such as our can help establish boundaries of plausible outcomes (eg, approximate ranges for educational costs, health care costs averted, or net benefits) that decision makers can apply locally. Finally, our findings regarding what works to enhance CPD effectiveness were limited by the paucity of studies and the wide variation in research questions.

## Conclusions

In this systematic review, CPD for drug prescribing was associated with reduced health care (drug) costs. The educational costs and cost-effectiveness of CPD varied widely. Several CPD instructional approaches (including educational outreach) were more effective but more costly than comparators.

## Notes

Supplement. **eBox.** Search Strategy and Definitions **eFigure 1.** Trial Flow **eTable 1.** Key Features of Individual Included Studies **eTable 2.** Key Features of Economic Methods and Reporting for Individual Included Studies **eFigure 2.** Cost-effectiveness and Cost-benefit (Education Costs and Clinical Outcomes): Dominance Ranking Matrix
eReferences

## References

[1] Ofori-Asenso R, Agyeman AA. Irrational use of medicines-a summary of key concepts. Pharmacy (Basel). 2016;4(4):35. doi:10.3390/pharmacy4040035 28970408 PMC5419375

[2] Liew TM, Lee CS, Goh SKL, Chang ZY. The prevalence and impact of potentially inappropriate prescribing among older persons in primary care settings: multilevel meta-analysis. Age Ageing. 2020;49(4):570-579. doi:10.1093/ageing/afaa057 32365168

[3] Liew TM, Lee CS, Goh Shawn KL, Chang ZY. Potentially inappropriate prescribing among older persons: a meta-analysis of observational studies. Ann Fam Med. 2019;17(3):257-266. doi:10.1370/afm.2373 31085530 PMC6827633

[4] Jungo KT, Streit S, Lauffenburger JC. Utilization and spending on potentially inappropriate medications by US older adults with multiple chronic conditions using multiple medications. Arch Gerontol Geriatr. 2021;93:104326. doi:10.1016/j.archger.2020.104326 33516154

[5] Fralick M, Bartsch E, Ritchie CS, Sacks CA. Estimating the use of potentially inappropriate medications among older adults in the United States. J Am Geriatr Soc. 2020;68(12):2927-2930. doi:10.1111/jgs.16779 32841366

[6] Davey P, Marwick CA, Scott CL, . Interventions to improve antibiotic prescribing practices for hospital inpatients. Cochrane Database Syst Rev. 2017;2:CD003543. doi:10.1002/14651858.CD003543.pub4 28178770 PMC6464541

[7] Taheri Moghadam S, Sadoughi F, Velayati F, Ehsanzadeh SJ, Poursharif S. The effects of clinical decision support system for prescribing medication on patient outcomes and physician practice performance: a systematic review and meta-analysis. BMC Med Inform Decis Mak. 2021;21(1):98. doi:10.1186/s12911-020-01376-8 33691690 PMC7944637

[8] Pearson SA, Moxey A, Robertson J, . Do computerised clinical decision support systems for prescribing change practice? a systematic review of the literature (1990-2007). BMC Health Serv Res. 2009;9:154. doi:10.1186/1472-6963-9-154 19715591 PMC2744674

[9] Arnold SR, Straus SE. Interventions to improve antibiotic prescribing practices in ambulatory care. Cochrane Database Syst Rev. 2005;(4):CD003539. doi:10.1002/14651858.CD003539.pub2 16235325 PMC7003679

[10] Grindrod KA, Patel P, Martin JE. What interventions should pharmacists employ to impact health practitioners’ prescribing practices? Ann Pharmacother. 2006;40(9):1546-1557. doi:10.1345/aph.1G300 16896025

[11] Ostini R, Hegney D, Jackson C, . Systematic review of interventions to improve prescribing. Ann Pharmacother. 2009;43(3):502-513. doi:10.1345/aph.1L488 19261953

[12] Brennan N, Mattick K. A systematic review of educational interventions to change behaviour of prescribers in hospital settings, with a particular emphasis on new prescribers. Br J Clin Pharmacol. 2013;75(2):359-372. doi:10.1111/j.1365-2125.2012.04397.x 22831632 PMC3579251

[13] Lu CY, Ross-Degnan D, Soumerai SB, Pearson SA. Interventions designed to improve the quality and efficiency of medication use in managed care: a critical review of the literature—2001-2007. BMC Health Serv Res. 2008;8:75. doi:10.1186/1472-6963-8-75 18394200 PMC2323373

[14] Pearson SA, Ross-Degnan D, Payson A, Soumerai SB. Changing medication use in managed care: a critical review of the available evidence. Am J Manag Care. 2003;9(11):715-731. 14626470

[15] Brown CA, Belfield CR, Field SJ. Cost effectiveness of continuing professional development in health care: a critical review of the evidence. BMJ. 2002;324(7338):652-655. doi:10.1136/bmj.324.7338.652 11895825 PMC84405

[16] Cook DA, Stephenson CR, Wilkinson JM, Maloney S, Baasch Thomas BL, Prokop LJ, . Costs and economic impacts of continuous professional development: a systematic scoping review. *Acad Med*. Published online August 24, 2021. 10.1097/ACM.0000000000004370 34432716

[17] Moher D, Liberati A, Tetzlaff J, Altman DG; PRISMA Group. Preferred reporting items for systematic reviews and meta-analyses: the PRISMA statement. Ann Intern Med. 2009;151(4):264-269, W64. doi:10.7326/0003-4819-151-4-200908180-00135 19622511

[18] Husereau D, Drummond M, Petrou S, ; CHEERS Task Force. Consolidated health economic evaluation reporting standards (CHEERS) statement. BMJ. 2013;346:f1049. doi:10.1136/bmj.f1049 23529982

[19] Sanders GD, Neumann PJ, Basu A, . Recommendations for conduct, methodological practices, and reporting of cost-effectiveness analyses: Second Panel on Cost-Effectiveness in Health and Medicine. JAMA. 2016;316(10):1093-1103. doi:10.1001/jama.2016.12195 27623463

[20] Neumann PJ, Sanders GD, Russell LB, Siegel JE, Ganiats TG, eds. Cost-Effectiveness in Health and Medicine, 2nd ed. Oxford University Press; 2016.

[21] Craig D, Rice S. NHS Economic Evaluation Database Handbook. Centre for Reviews and Dissemination; 2007.

[22] Levin HM, McEwan PJ, Belfield CR, Bowden AB, Shand RD. Economic Evaluation in Education: Cost-Effectiveness and Benefit-Cost Analysis. 3rd ed. Sage Publications; 2017.

[23] Cook DA, Reed DA. Appraising the quality of medical education research methods: the Medical Education Research Study Quality Instrument and the Newcastle-Ottawa Scale-Education. Acad Med. 2015;90(8):1067-1076. doi:10.1097/ACM.0000000000000786 26107881

[24] Drummond MF, Jefferson TO; The BMJ Economic Evaluation Working Party. Guidelines for authors and peer reviewers of economic submissions to the BMJ. BMJ. 1996;313(7052):275-283. doi:10.1136/bmj.313.7052.275 8704542 PMC2351717

[25] Anderson R. Systematic reviews of economic evaluations: utility or futility? Health Econ. 2010;19(3):350-364. doi:10.1002/hec.1486 19378354

[26] Higgins JPT, Green S. *Cochrane Handbook for Systematic Reviews of Interventions 5.1.0.* Updated March 2011. Accessed May 31, 2018. http://handbook-5-1.cochrane.org/

[27] Gomersall JS, Jadotte YT, Xue Y, Lockwood S, Riddle D, Preda A. Conducting systematic reviews of economic evaluations. Int J Evid Based Healthc. 2015;13(3):170-178. doi:10.1097/XEB.0000000000000063 26288063

[28] Shields GE, Elvidge J. Challenges in synthesising cost-effectiveness estimates. Syst Rev. 2020;9(1):289. doi:10.1186/s13643-020-01536-x 33298168 PMC7727163

[29] Nixon J, Khan KS, Kleijnen J. Summarising economic evaluations in systematic reviews: a new approach. BMJ. 2001;322(7302):1596-1598. doi:10.1136/bmj.322.7302.1596 11431306 PMC1120631

[30] Moleski RJ, Andriole VT. Role of the infectious disease specialist in containing costs of antibiotics in the hospital. Rev Infect Dis. 1986;8(3):488-493. doi:10.1093/clinids/8.3.488 3726398

[31] Soumerai SB, Avorn J. Economic and policy analysis of university-based drug “detailing”. Med Care. 1986;24(4):313-331. doi:10.1097/00005650-198604000-00003 3083161

[32] Landgren FT, Harvey KJ, Mashford ML, Moulds RF, Guthrie B, Hemming M. Changing antibiotic prescribing by educational marketing. Med J Aust. 1988;149(11-12):595-599. doi:10.5694/j.1326-5377.1988.tb120797.x 3200183

[33] Raisch DW, Bootman JL, Larson LN, McGhan WF. Improving antiulcer agent prescribing in a health maintenance organization. Am J Hosp Pharm. 1990;47(8):1766-1773. doi:10.1093/ajhp/47.8.1766 2389782

[34] Friis H, Bro F, Mabeck CE, Vejlsgaard R. Changes in prescription of antibiotics in general practice in relation to different strategies for drug information. Dan Med Bull. 1991;38(4):380-382. 1914537

[35] Stuart ME, Handley MA, Chamberlain MA, Wallach RW, Penna PM, Stergachis A. Successful implementation of a guideline program for the rational use of lipid-lowering drugs. HMO Pract. 1991;5(6):198-204. 10115851

[36] Hadbavny AM, Hoyt JW. Promotion of cost-effective benzodiazepine sedation. Am J Hosp Pharm. 1993;50(4):660-661. 8470677

[37] Weir B. Antibiotic prophylaxis in cesarean section: use of cost per case comparison to influence prescribing practices. Hosp Formul. 1993;28(3):285-286, 289-290. 10124450

[38] Zimmerman DR, Collins TM, Lipowski EE, Sainfort F. Evaluation of a DUR intervention: a case study of histamine antagonists. Inquiry. 1994;31(1):89-101. 7909535

[39] Ziskind AA, Portelli J, Rodriguez S, . Successful use of education and cost-based feedback strategies to reduce physician utilization of low-osmolality contrast agents in the cardiac catheterization laboratory. Am J Cardiol. 1994;73(16):1219-1221. doi:10.1016/0002-9149(94)90187-2 8203344

[40] Bausch J. The pharmacotherapy circle—a promising way for improving quality in primary medical care. Article in German. Z Arztl Fortbild (Jena). 1995;89(4):406-414. 7571744

[41] Schectman JM, Schroth WS, Elinsky EG, Ott JE. The effect of education and drug samples on antihistamine prescribing costs in an HMO. HMO Pract. 1996;10(3):119-122. 10160286

[42] von Ferber L, Bausch J, Schubert I, Köster I, Ihle P. Drug therapy courses for family physicians—advanced education in pharmacotherapy. Article in German. Z Arztl Fortbild Qualitatssich. 1997;91(8):767-772. 9487632

[43] Boreen D, Juge D, Stahl J, Torborg S. Effects of a physician education program on prescribing of HMG-CoA reductase inhibitors. Formulary. 1998;33:802-809.

[44] Use physician education to cut prescription drug costs. Capitation Manag Rep. 1999;6(7):100-103, 97. 10539514

[45] Hux JE, Melady MP, DeBoer D. Confidential prescriber feedback and education to improve antibiotic use in primary care: a controlled trial. CMAJ. 1999;161(4):388-392. 10478162 PMC1230539

[46] McNulty CA, Kane A, Foy CJ, Sykes J, Saunders P, Cartwright KA. Primary care workshops can reduce and rationalize antibiotic prescribing. J Antimicrob Chemother. 2000;46(3):493-499. doi:10.1093/jac/46.3.493 10980182

[47] Valori RM, Brown CM, Strangeways P, Bradburn M. Reducing community dyspepsia drug costs: a controlled trial. Gut. 2001;49(4):495-501. doi:10.1136/gut.49.4.495 11559645 PMC1728459

[48] Watson M, Gunnell D, Peters T, Brookes S, Sharp D. Guidelines and educational outreach visits from community pharmacists to improve prescribing in general practice: a randomised controlled trial. J Health Serv Res Policy. 2001;6(4):207-213. doi:10.1258/1355819011927503 11685784

[49] Bell N. Antibiotic resistance: the Iowa experience. Am J Manag Care. 2002;8(11):988-994. 12437313

[50] Bernal-Delgado E, Galeote-Mayor M, Pradas-Arnal F, Peiró-Moreno S. Evidence based educational outreach visits: effects on prescriptions of non-steroidal anti-inflammatory drugs. J Epidemiol Community Health. 2002;56(9):653-658. doi:10.1136/jech.56.9.653 12177080 PMC1732253

[51] Dobscha SK, Anderson TA, Hoffman WF, . Strategies to decrease costs of prescribing selective serotonin reuptake inhibitors at a VA Medical Center. Psychiatr Serv. 2003;54(2):195-200. doi:10.1176/appi.ps.54.2.195 12556600

[52] Lutters M, Harbarth S, Janssens J-P, . Effect of a comprehensive, multidisciplinary, educational program on the use of antibiotics in a geriatric university hospital. J Am Geriatr Soc. 2004;52(1):112-116. doi:10.1111/j.1532-5415.2004.52019.x 14687324

[53] Madridejos-Mora R, Amado-Guirado E, Pérez-Rodríguez MT. Effectiveness of the combination of feedback and educational recommendations for improving drug prescription in general practice. Med Care. 2004;42(7):643-648. doi:10.1097/01.mlr.0000129495.43422.58 15213488

[54] Simon SR, Majumdar SR, Prosser LA, . Group versus individual academic detailing to improve the use of antihypertensive medications in primary care: a cluster-randomized controlled trial. Am J Med. 2005;118(5):521-528. doi:10.1016/j.amjmed.2004.12.023 15866255

[55] Chazan B, Turjeman RBZ, Frost Y, . Antibiotic consumption successfully reduced by a community intervention program. Isr Med Assoc J. 2007;9(1):16-20. 17274349

[56] Siriwardena AN, Fairchild P, Gibson S, Sach T, Dewey M. Investigation of the effect of a countywide protected learning time scheme on prescribing rates of ramipril: interrupted time series study. Fam Pract. 2007;24(1):26-33. doi:10.1093/fampra/cml051 17052988

[57] Apisarnthanarak A, Yatrasert A, Mundy LM; Thammasat University Antimicrobial Stewardship Team. Impact of education and an antifungal stewardship program for candidiasis at a Thai tertiary care center. Infect Control Hosp Epidemiol. 2010;31(7):722-727. doi:10.1086/653616 20497042

[58] Niquille A, Ruggli M, Buchmann M, Jordan D, Bugnon O. The nine-year sustained cost-containment impact of swiss pilot physicians-pharmacists quality circles. Ann Pharmacother. 2010;44(4):650-657. doi:10.1345/aph.1M537 20215496

[59] Lopez-Picazo JJ, Ruiz JC, Sanchez JF, Ariza A, Aguilera B. A randomized trial of the effectiveness and efficiency of interventions to reduce potential drug interactions in primary care. Am J Med Qual. 2011;26(2):145-153. doi:10.1177/1062860610380898 21403177

[60] Qureshi NA, Neyaz Y, Khoja T, Magzoub MA, Haycox A, Walley T. Effectiveness of three interventions on primary care physicians’ medication prescribing in Riyadh City, Saudi Arabia [erratum appears in *East Mediterr Health J*. 2011;17(3):249]. East Mediterr Health J. 2011;17(2):172-179. doi:10.26719/2011.17.2.172 21735954

[61] Weiss K, Blais R, Fortin A, Lantin S, Gaudet M. Impact of a multipronged education strategy on antibiotic prescribing in Quebec, Canada. Clin Infect Dis. 2011;53(5):433-439. doi:10.1093/cid/cir409 21791439

[62] del Arco A, Tortajada B, de la Torre J, . Results of a counselling programme in antibiotic treatment in a secondary hospital. Article in Spanish. Rev Esp Quimioter. 2011;24(2):96-98. 21667002

[63] Butler CC, Simpson SA, Dunstan F, . Effectiveness of multifaceted educational programme to reduce antibiotic dispensing in primary care: practice based randomised controlled trial. BMJ. 2012;344:d8173. doi:10.1136/bmj.d8173 22302780 PMC3270575

[64] Dormuth CR, Carney G, Taylor S, Bassett K, Maclure M. A randomized trial assessing the impact of a personal printed feedback portrait on statin prescribing in primary care. J Contin Educ Health Prof. 2012;32(3):153-162. doi:10.1002/chp.21140 23008077

[65] Le Corvoisier P, Renard V, Roudot-Thoraval F, . Long-term effects of an educational seminar on antibiotic prescribing by GPs: a randomised controlled trial. Br J Gen Pract. 2013;63(612):e455-e464. doi:10.3399/bjgp13X669176 23834882 PMC3693802

[66] Holuby RS, Pellegrin KL, Barbato A, Ciarleglio A. Recruitment of rural healthcare professionals for live continuing education. Med Educ Online. 2015;20:28958. doi:10.3402/meo.v20.28958 26549047 PMC4637333

[67] Pechlivanoglou P, Wieringa JE, de Jager T, Postma MJ. The effect of financial and educational incentives on rational prescribing. a state-space approach. Health Econ. 2015;24(4):439-453. doi:10.1002/hec.3030 24519732

[68] Tolsgaard MG, Cook DA. New roles for cost as an outcome: opportunities and challenges. Med Educ. 2017;51(7):680-682. doi:10.1111/medu.13328 28722187

[69] Maloney S, Cook DA, Golub R, . AMEE Guide No. 123—how to read studies of educational costs. Med Teach. 2019;41(5):497-504. doi:10.1080/0142159X.2018.1552784 30794756

[70] Walsh K. Cost-effectiveness in Medical Education. Radcliffe; 2010.

[71] Foo J, Cook DA, Walsh K, . Cost evaluations in health professions education: a systematic review of methods and reporting quality. Med Educ. 2019;53(12):1196-1208. doi:10.1111/medu.13936 31402515

[72] Zendejas B, Wang AT, Brydges R, Hamstra SJ, Cook DA. Cost: the missing outcome in simulation-based medical education research: a systematic review. Surgery. 2013;153(2):160-176. doi:10.1016/j.surg.2012.06.025 22884087

