# Supplementary material for: Cost-effectiveness and Economic Benefit of Continuous Professional Development for Drug Prescribing: A Systematic Review
Source: JAMA Netw Open. 2022 Jan 26;5(1):e2144973. doi: 10.1001/jamanetworkopen.2021.44973 (PMC8792887; doi:10.1001/jamanetworkopen.2021.44973)
Supplement: Supplement. — eBox. Search Strategy and Definitions eFigure 1. Trial Flow eTable 1. Key Features of Individual Included Studies eTable 2. Key Features of Economic Methods and Reporting for Individual Included Studies eFigure 2. Cost-effectiveness and Cost-benefit (Education Costs and Clinical Outcomes): Dominance Ranking Matrix eReferences [file jamanetwopen-e2144973-s001.pdf]

Supplementary Online Content

Cook DA, Stephenson CR, Wilkinson JM, Maloney S, Foo J. Cost-effectiveness and economic benefit of continuous professional development for drug prescribing: a systematic review. *JAMA Netw Open*. 2022;5(1):e2144973. doi:10.1001/jamanetworkopen.2021.44973

eBox. Search Strategy and Definitions
eFigure 1. Trial Flow
eTable 1. Key Features of Individual Included Studies
eTable 2. Key Features of Economic Methods and Reporting for Individual Included Studies
eFigure 2. Cost-effectiveness and Cost-benefit (Education Costs and Clinical Outcomes): Dominance Ranking Matrix
eReferences

This supplementary material has been provided by the authors to give readers additional information about their work.

© 2022 Cook DA et al. *JAMA Network Open.*

eBox. Search strategy and definitions

Search strategy

We used OVID to search the following databases, on April 23, 2020, using the search strategy below:

- Ovid MEDLINE(R) and Epub Ahead of Print, In-Process & Other Non-Indexed Citations and Daily 1946 to April 22, 2020
- Embase 1974 to 2020 April 22,
- APA PsycInfo 1806 to April Week 2 2020,
- EBM Reviews - Cochrane Central Register of Controlled Trials March 2020,
- EBM Reviews - Cochrane Database of Systematic Reviews 2005 to April 22, 2020,

We used the following search strategy:

#	Searches	Results
1	"education medical continuing".hw.	25414
2	exp Education, Continuing/	93064
3	((continuing or continued or continuous or postgraduate) adj3 (health or medical or professional) adj3 (education or development)).ti,ab,hw,kw.	52005
4	2 or 3	115168
5	exp Physicians/	914857
6	(Allergist* or Anaesthesiologist* or Andrologist* or Anesthesiologist* or cardiologist* or clinician* or dermatologist* or doctor* or endocrinologist* or Epidemiologist* or feldsher* or fellow or fellows or gastroenterologist* or gastrologist* or Geneticist* or geriatrician* or gerontologist* or Gynecologist* or Hematologist* or Hepatologist* or hospitalist* or Immunologist* or "Infectious Disease Specialist*" or "Internal Medicine Specialist*" or Internist* or medical or Microbiologist* or Neonatologist* or nephrologist* or neurologist* or Neurophysiologist* or neurosurgeon* or obstetrician* or oncologist* or Ophthalmologist* or Orthopedist* or otolaryngologist* or pathologist* or Pediatrician* or physician* or Physiologist* or podiatrist* or practitioner* or provider* or Psychiatrist* or Pulmonologist* or radiologist* or resident* or rheumatologist* or surgeon* or urologist*).ti,ab,hw,kw.	8271061
7	5 or 6	8273613
8	(4 and 7) or 1	72193
9	exp *Economics, Medical/ or ("education medical continuing".hw. and exp Economics, Medical/)	260969
10	exp Cost-Benefit Analysis/	170873
11	exp "cost effectiveness analysis"/	229704
12	"Costs and Cost Analysis"/	119060
13	exp "Cost Savings"/	79853
14	((economic* adj3 (benefit* or saving* or factor* or improv* or value)) or (value adj1 money) or budget* or "containing cost" or "containing costs" or "controlling cost" or "controlling costs" or "cost containment" or "cost control" or "cost effectiveness" or "cost saving*" or "Cost-Benefit" or "cost-benefits" or costs or efficiency or expenditure* or monetary or "return on investment" or revenue).ti,ab,hw,kw.	2254760
15	9 or 10 or 11 or 12 or 13 or 14	2420662
16	(referral adj3 (pattern* or increas* or improv*)).ti,ab,hw,kw.	14222
17	((continuing or continued or continuous or postgraduate) adj3 (health or medical or professional) adj3 (education or development) adj5 (value or benefit or benefits or (improv* adj5 (care or outcome*)))).ti,ab,hw,kw.	509
18	15 or 16 or 17	2433953
19	8 and 18	4584
20	limit 19 to (dissertation abstract or editorial or erratum or note or addresses or autobiography or bibliography or biography or blogs or comment or dictionary or directory or interactive tutorial or interview or lectures or legal cases or legislation or news or newspaper article or overall or patient education handout or periodical index or portraits or published erratum or video-audio media or webcasts) [Limit not valid in PsycINFO,CCTR,CDSR,Embase,Ovid	279

© 2022 Cook DA et al. *JAMA Network Open.*

	MEDLINE(R),Ovid MEDLINE(R) Daily Update,Ovid MEDLINE(R) In-Process,Ovid MEDLINE(R) Publisher; records were retained]	
21	19 not 20	4305
22	remove duplicates from 21	3399

Further deduplication using a library protocol identified 46 duplicates, and then additional de-duplication in EndNote identified 15 additional duplicate articles, 1 full reprint, and 2 concurrently-published policy statements. These 64 articles were removed, leaving 3335 articles to be screened from this search strategy.

In addition, we found 3 articles from review of reference lists, for a total of 3338 articles screened.

Definitions of key terms

- *Physician continuing professional development (CPD)*: Activities intended to promote or measure the clinical knowledge/skills of physicians in independent medical practice through a) courses or assessments delivered in any modality or venue, whether or not continuing medical education (CME) credit is awarded, or b) self-directed learning or self-assessment activities for which CME credit is awarded.
    - This included programs that combined a practice-focused (non-education) intervention with a distinct training component.
    - This excluded self-directed learning activities that did not receive CME credit, activities intended to develop non-clinical skills, and activities solely targeting physicians in postgraduate training (i.e., residents).
- *Comparative economic evaluation*: A study comparing a) economic metrics (monetary cost [inputs] or economic impact [outcomes]) of two activities, or b) economic metrics and consequences (effectiveness or non-economic benefits or harms) of one activity.
    - This included full economic evaluations (including cost-effectiveness analysis, cost-benefit analysis, and cost-utility analysis), partial economic evaluations (comparisons of economic metrics for two activities), and cost outcome descriptions (comparisons of economic metrics and consequences for one activity).
- *Cost*: The value of opportunity forgone as a result of engaging resources in an activity.
    - This included monetary costs associated with course planning and implementation, and monetary and non-monetary faculty and learner time or lost revenue.
- *Economic impact*: a) a monetary expression of any outcome (monetary impact; e.g., direct costs of diagnosis or treatment, and indirect costs to society of poor health or longer life) or b) an economic utility (preference-based health outcome, such as quality-adjusted life year [QALY]).

© 2022 Cook DA et al. *JAMA Network Open.*

eFigure 1. Trial flow

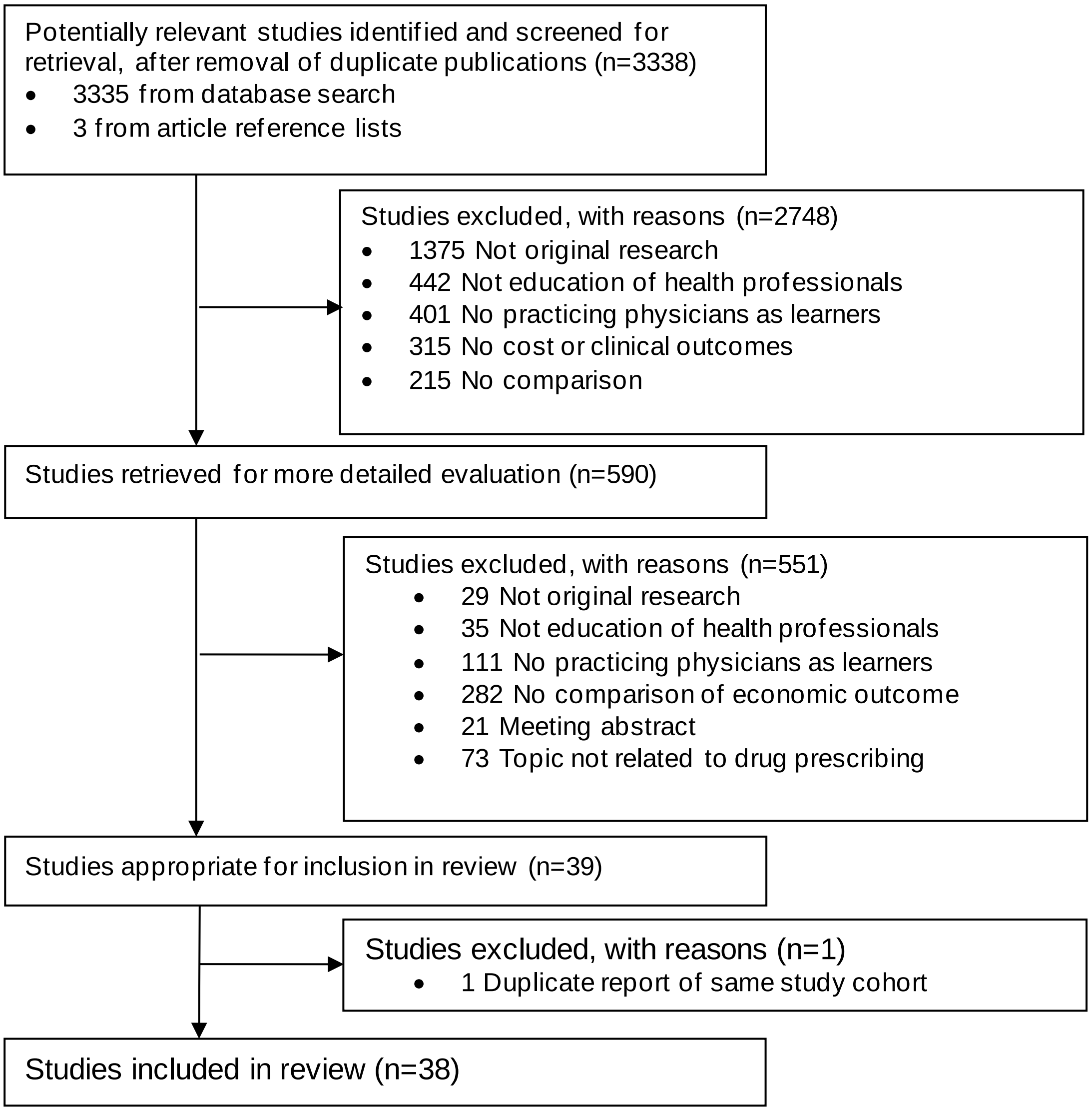

© 2022 Cook DA et al. *JAMA Network Open.*

eTable 1. Key features of individual included studies

Author, year	Participants specialty [Nonphysician participants]	Geographic origin (Funding)	Design (Comparison)	Education target	Education approach	Education cost: Quantity; Pricing	Drug cost: Quantity; Pricing (Time, wks)	Outcome validation	MERSQI total score
Moleski et al,[1] 1986	Surg	USA (Ind)	PP1 (NI)	Abx	LgGrp, Paper, SmGrp		NR; NR (52)		6
Soumerai et al,[2] 1986	Other/Vague	USA (Govt)	RCT (NI, CPD)	Abx, Analg, N/Psych	Paper, SmGrp	NR; NR	Claim; Claim (39)		11.5
Landgren et al,[3] 1988	All	Oceania (Govt)	NR2 (NI)	Abx	LgGrp, Paper, Video	NR; NR	SelfRpt; Other (3)	Cont	11.5
Raisch et al,[4] 1990	Other/Vague, [NPPA]	USA (NR-Cap)	RCT (NI, CPD)	GI	Case, Paper, SmGrp		Chart; AWP (4)	IS	14
Friis et al,[5] 1991	FM/IM	Denmark (Org)	NR2 (NI)	Abx	LgGrp, Paper		SelfRpt; AWP (1)		8.5
Stuart et al,[6] 1991	FM/IM, [Pharm]	USA (NR-Cap)	NR2 (NI)	Lipid	Audit, Case, LgGrp, Paper, SmGrp		NR; AWP (52)		5.5
Hadbavny et al,[7] 1993	CritCare	USA (NR)	PP1 (NI)	N/Psych	Paper, SmGrp		NR; NR (NR)		3
Weir,[8] 1993	ObGyn	USA (NR)	PP1 (NI)	Abx	Audit, LgGrp, Paper		Chart; NR (4)		6
Zimmerman et al,[9] 1994	Other/Vague	USA (NR)	NR2 (NI)	H1	Audit, Paper	NR; Estim	Claim; Claim (52)		11.5
Ziskind et al,[10] 1994	IntMedSp	USA (NR)	PP1 (NI)	Dye	Audit, Paper		Claim; Claim (13)		10.5
Bausch,[11] 1995	FM/IM	Germany (NR)	PP1 (NI)	Mix	SmGrp		NR; NR (13)		9.5
Schectman et al,[12] 1996	FM/IM, [NPPA]	USA (NR-Cap)	NR2 (NI)	H1	Clinic, Paper		Claim; Claim (35)	IS	13
von Ferber et al,[13] 1997	FM/IM	Germany (Ind)	PP1 (NI)		Audit, SmGrp		NR; NR (13)		8

© 2022 Cook DA et al. *JAMA Network Open.*

Author, year	Participants specialty [Nonphysician participants]	Geographic origin (Funding)	Design (Comparison)	Education target	Education approach	Education cost: Quantity; Pricing	Drug cost: Quantity; Pricing (Time, wks)	Outcome validation	MERSQI total score
Boreen et al,[14] 1998	FM/IM	USA (NR-Cap)	PP1 (NI)	Lipid	Audit, LgGrp, SmGrp		Claim; Claim (39)		9
Hux et al,[15] 1999	FM/IM	Canada (NR)	RCT (NI)	Abx	Audit, Paper		NR; Claim (26)	Cont	14
Unnamed,[16] 1999	Other/Vague	USA (NR-Cap)	NR2 (NI)	Mix	Audit, SmGrp		NR; Claim (NR)		3.5
McNulty et al,[17] 2000	FM/IM	UK (NR)	NR2 (NI)	Abx	Audit, SmGrp	NR; NR	Claim; Claim (17)	Cont	11.5
Valori et al,[18] 2001	FM/IM, [PG]	UK (NR)	NR2 (NI)	GI	LgGrp, Paper	NR; NR	NR; Claim (143)		11.5
Watson et al,[19] 2001	FM/IM	UK (Govt)	RCT (NI, CPD)	Analg	Paper, SmGrp	NR; NR	Claim; Claim (12)	Cont, IS	14.5
Bell,[20] 2002	FM/IM, Peds, [Pharm], [NPPA]	USA (Ind)	PP1 (NI)	Abx	Audit, Paper, SmGrp		NR; NR (NR)		9
Bernal-Delgado et al,[21] 2002	FM/IM	Spain (Govt)	RCT (NI, CPD)	Analg	Paper, SmGrp		Claim; Claim (26)		11.5
Dobscha et al,[22] 2003	FM/IM, Other/Vague, [PG], [NPPA]	USA (Govt)	PP1 (NI)	N/Psych	Audit, Clinic, Internet, Paper		Claim; Claim (NR)	Cont, IS	10
Lutters et al,[23] 2004	FM/IM, [PG]	Switz. (NR)	PP1 (NI)	Abx	Audit, LgGrp, Paper		Chart; AWP (NR)		11.5
Madridejos-Mora et al,[24] 2004	FM/IM	Spain (Org)	RCT (NI)	Mix	Audit, Paper, SmGrp		Claim; NR (17)		14
Simon et al,[25] 2005	FM/IM, [NPPA]	USA (NR)	RCT (CPD)	CV	Paper, SmGrp	NR; NR	Claim; Claim (9)		12.5
Chazan et al,[26] 2007	FM/IM, [Pharm], [RN]	Israel (NR)	RCT (CPD)	Abx	LgGrp, Paper		Claim; NR (13)		11.5
Siriwardena et al,[27] 2007	FM/IM, [RN]	UK (NR)	PP1 (NI)	CV	LgGrp, SmGrp	NR; Log	Claim; AWP (12)		10.5

© 2022 Cook DA et al. *JAMA Network Open.*

Author, year	Participants specialty [Nonphysician participants]	Geographic origin (Funding)	Design (Comparison)	Education target	Education approach	Education cost: Quantity; Pricing	Drug cost: Quantity; Pricing (Time, wks)	Outcome validation	MERSQI total score
Apisarnthanarak et al,[28] 2010	All, [PG], [MS]	Thai (Local)	PP1 (NI)	Abx	Audit, Clinic, LgGrp		NR; Claim (78)	Cont	12
Niquille et al,[29] 2010	FM/IM	Switz. (Ind)	NR2 (NI)	Mix	Audit, SmGrp		Claim; Claim (52)		9.5
del Arco et al,[30] 2011	FM/IM, IntMedSp, OtherMed, Surg	Spain (NR)	PP1 (NI)	Abx	SmGrp		NR; NR (52)		8.5
Lopez-Picazo et al,[31] 2011	FM/IM	Spain (Govt)	RCT (NI, CPD)	Errors	Audit, LgGrp, SmGrp	Log; Log, Publish		Cont	14
Qureshi et al,[32] 2011	FM/IM	S.Arabia (NR)	NR2 (NI, CPD)	Errors	LgGrp, Paper, SmGrp	Log; Log			11
Weiss et al,[33] 2011	All, [PG], [Pharm]	Canada (Ind)	NR2 (NI)	Abx	Paper		Claim; Claim (151)		11.5
Butler et al,[34] 2012	FM/IM	UK (Govt)	RCT (NI)	Abx	Audit, Internet, Paper, SmGrp, Video	Log; Log, Publish	Claim; BNP (52)	Cont	16
Dormuth et al,[35] 2012	FM/IM	Canada (Govt)	RCT (NI)	Lipid	Audit, Paper		Claim; Claim (52)	Cont, IS	14.5
Le Corvoisier et al,[36] 2013	FM/IM	France (Govt)	RCT (NI, CPD)	Abx	Audit, LgGrp, Pt/Sim, SmGrp		Claim; Other (13)		14
Holuby et al,[37] 2015	FM/IM, [Pharm], [RN]	USA (Govt)	NR2 (CPD)	Errors		NR; NR			7
Pechlivanoglou et al,[38] 2015	All	Nether. (NR)	NR2 (NI)	Mix	Clinic, Paper		Claim; Claim (182)	Cont	11.5

Abbreviations: NR = not reported; Educ = education.
Participants: All = all specialties; FM/IM = family / internal / general medicine; IntMedSp = internal medicine subspecialty; OtherMed = other medical specialty; ObGyn = obstetrics/gynecology; Peds = pediatrics or pediatric subspecialty; Surg = surgery. All studies enrolled physicians in independent practice; additional health professions learners are noted in [brackets]: [MS] = medical students; [NPPA] = nurse practitioner or physician assistant, or student; [PG] = physicians in postgraduate training; [Pharm] = pharmacist or pharmacy student; [RN] = nurse or nursing student.

© 2022 Cook DA et al. *JAMA Network Open.*

Geographic origin: Nether. = The Netherlands; Switz. = Switzerland; Thai. = Thailand; UK = United Kingdom; USA = United States of America.
Funding: Ind = industry; Local = local institutional support (grant or no funding); NR = not reported; NR-Cap = not reported, authors employed by a capitated health plan; Org = philanthropic or professional organization.
Design (Comparison): RCT = randomized controlled trial; NR2 = non-randomized comparison (≥2 groups); PP1 = 1-group pre/post-intervention comparison or time series; CPD = comparison with alternate CPD (educational) intervention; NI = comparison with no intervention.
Education target: Abx = specific drug (antibiotics); Analg = specific drug (analgesic [nonsteroidal anti-inflammatory or opioid]); CV = specific drug (cardiovascular, hypertension); Dye = specific drug (radiocontrast dye); Errors = prevention of prescribing errors in general; GI = specific drug (gastrointestinal); H1= specific drug (allergy [H1] antihistamines); Lipid = specific drug (antihyperlipidemics); Mix = improvement of prescribing for a range of drugs; N/Psych = specific drug (neurological/psychiatric).
Education approach: Audit = audit and feedback; Case = case-based learning (practice problems); Clinic = clinical practice change; Internet = Internet-based instruction; LgGrp = large group (lecture); Paper = paper materials; Pt/Sim = practice with real or simulated patient; SmGrp = small group (includes 1-on-1 educational outreach); Video = video clip.
Education cost (resource quantity and unit price): Log = log of actual resources used or actual costs; Estim = estimated from program budget; Publish = published fee schedule.
Drug cost (resource quantity, unit price, time): AWP = average wholesale price; BNP = British National Formulary; Chart = manual review of medical record; Claim = claims database or billing codes; Other = other publication; SelfRpt = reported by clinician. Time = duration of data collection post-education (weeks); NR = not reported.
Outcome validation: Cont = evidence of content, IS = evidence of internal structure.
MERSQI = Medical Education Research Study Quality Instrument;[39] total score range 5 to 18 possible.

© 2022 Cook DA et al. *JAMA Network Open.*

eTable 2. Key features of economic methods and reporting for individual included studies

Author, year	8 Effectiveness outcomes source	9 Design of effectiveness study	16 Resources quantified (education)	16 Resources quantified (health care)	17 Quantity method (education)	17 Pricing method (education)	17 Quantity method (health care)	17 Pricing method (health care)	18 Currency	22 Time horizon	23 Discount rate defined	24/25 Discount rate justified	27 Sensitivity analysis done	30 Relevant comparison	31 Incremental analysis	32 Outcomes disaggregated
**Studies of cost-effectiveness and net benefits**																
Soumerai et al,[2] 1986	1	1	1		0	0			1	1	1	1	1	1	0	1
Landgren et al,[3] 1988	1	1	0		0	0			1	1	0	0	0	0	0	1
Zimmerman et al,[9] 1994	1	1	0		0	1			1	1	0	0	0	0	1	1
McNulty et al,[17] 2000	1	1	0		0	0			1	1	0	0	0	0	0	1
Valori et al,[18] 2001	0	1	0		0	0			1	1	0	0	0	0	0	1
Watson et al,[19] 2001	1	1	0		0	0			1	1	0	0	1	1	1	1
Simon et al,[25] 2005	1	1	0		0	0			1	1	0	0	0	1	0	1
Siriwardena et al,[27] 2007	1	1	1		0	1			1	1	0	0	0	0	1	1

**Author, year**	**8 Effectiveness outcomes source**	**9 Design of effectiveness study**	**16 Resources quantified (education)**	**16 Resources quantified (health care)**	**17 Quantity method (education)**	**17 Pricing method (education)**	**17 Quantity method (health care)**	**17 Pricing method (health care)**	**18 Currency**	**22 Time horizon**	**23 Discount rate defined**	**24/25 Discount rate justified**	**27 Sensitivity analysis done**	**30 Relevant comparison**	**31 Incremental analysis**	**32 Outcomes disaggregated**
Lopez-Picazo et al,[31] 2011	1	1	1		1	1			1	1	0	0	0	1	1	1
Qureshi et al,[32] 2011	1	1	1		1	1			1	0	0	0	0	1	0	1
Butler et al,[34] 2012	1	1	1		1	1			1	1	0	1	0	0	1	1
Holuby et al,[37] 2015	1	1	0		0	0			1	1	0	0	0	1	1	1
Number of studies with feature present (of 12)	***11***	***12***	***5***		***3***	***5***			***12***	***11***	***1***	***2***	***2***	***6***	***6***	***12***
**Studies of economic effects**																
Moleski et al,[1] 1986	0	1		1			0	0	1	1	0	0	0	0	0	1
Soumerai et al,[2] 1986	1	1		1			1	1	1	1	1	1	1	1	0	1
Landgren et al,[3] 1988	1	1		1			1	1	1	1	0	0	0	0	0	1
Raisch et al,[4] 1990	1	1		1			1	1	1	1	1	1	0	1	0	1

© 2022 Cook DA et al. *JAMA Network Open.*

**Author, year**	**8 Effectiveness outcomes source**	**9 Design of effectiveness study**	**16 Resources quantified (education)**	**16 Resources quantified (health care)**	**17 Quantity method (education)**	**17 Pricing method (education)**	**17 Quantity method (health care)**	**17 Pricing method (health care)**	**18 Currency**	**22 Time horizon**	**23 Discount rate defined**	**24/25 Discount rate justified**	**27 Sensitivity analysis done**	**30 Relevant comparison**	**31 Incremental analysis**	**32 Outcomes disaggregated**
Friis et al,[5] 1991	1	1		1			1	1	1	1	0	0	0	0	0	1
Stuart et al,[6] 1991	0	1		0			0	1	1	1	0	0	0	0	0	1
Hadbavny et al,[7] 1993	0	1		1			0	0	1	0	0	0	0	0	0	1
Weir,[8] 1993	1	1		1			1	0	1	1	0	0	0	0	0	1
Zimmerman et al,[9] 1994	1	1		1			1	1	1	1	0	0	0	0	1	1
Ziskind et al,[10] 1994	1	1		1			1	1	1	1	0	0	0	0	0	1
Bausch,[11] 1995	0	1		0			0	0	1	1	0	0	0	0	0	1
Schectman et al,[12] 1996	1	1		1			1	1	1	1	0	0	0	0	0	1
von Ferber et al,[13] 1997	0	1		0			0	0	1	1	0	0	0	0	0	1
Boreen et al,[14] 1998	1	1		1			1	1	1	1	0	0	0	0	0	1
Hux et al,[15] 1999	0	1		0			0	1	1	1	0	0	0	0	0	1
Unnamed,[16] 1999	0	1		0			0	1	1	0	0	0	0	0	0	1

© 2022 Cook DA et al. *JAMA Network Open.*

Author, year	8 Effectiveness outcomes source	9 Design of effectiveness study	16 Resources quantified (education)	16 Resources quantified (health care)	17 Quantity method (education)	17 Pricing method (education)	17 Quantity method (health care)	17 Pricing method (health care)	18 Currency	22 Time horizon	23 Discount rate defined	24/25 Discount rate justified	27 Sensitivity analysis done	30 Relevant comparison	31 Incremental analysis	32 Outcomes disaggregated
McNulty et al,[17] 2000	1	1		1			1	1	1	1	0	0	0	0	0	1
Valori et al,[18] 2001	0	1		0			0	1	1	1	0	0	0	0	0	1
Watson et al,[19] 2001	1	1		1			1	1	1	1	0	0	1	1	1	1
Bell,[20] 2002	0	1		0			0	0	1	0	0	0	0	0	0	1
Bernal-Delgado et al,[21] 2002	1	1		1			1	1	0	1	0	0	0	1	0	1
Dobscha et al,[22] 2003	1	1		1			1	1	1	0	0	0	0	0	0	1
Lutters et al,[23] 2004	1	1		1			1	1	1	0	0	0	0	0	0	1
Madridejos-Mora et al,[24] 2004	1	1		1			1	0	1	1	0	0	0	0	0	1
Simon et al,[25] 2005	1	1		1			1	1	1	1	0	0	0	1	0	1
Chazan et al,[26] 2007	1	1		1			1	0	1	1	0	0	0	1	0	1
Siriwardena et al,[27] 2007	1	1		1			1	1	1	1	0	0	0	0	1	1

© 2022 Cook DA et al. *JAMA Network Open.*

**Author, year**	**8 Effectiveness outcomes source**	**9 Design of effectiveness study**	**16 Resources quantified (education)**	**16 Resources quantified (health care)**	**17 Quantity method (education)**	**17 Pricing method (education)**	**17 Quantity method (health care)**	**17 Pricing method (health care)**	**18 Currency**	**22 Time horizon**	**23 Discount rate defined**	**24/25 Discount rate justified**	**27 Sensitivity analysis done**	**30 Relevant comparison**	**31 Incremental analysis**	**32 Outcomes disaggregated**
Apisarnthanarak et al,[28] 2010	0	1		0			0	1	1	1	0	0	0	0	0	1
Niquille et al,[29] 2010	1	1		1			1	1	1	1	0	0	0	0	0	1
Weiss et al,[33] 2011	1	1		1			1	1	1	1	0	0	0	0	0	1
del Arco et al,[30] 2011	0	1		0			0	0	1	1	0	0	0	0	0	1
Butler et al,[34] 2012	1	1		1			1	1	1	1	0	1	0	0	1	1
Dormuth et al,[35] 2012	1	1		1			1	1	1	1	0	0	1	0	0	1
Le Corvoisier et al,[36] 2013	1	1		1			1	1	1	1	0	0	0	1	0	1
Pechlivanoglou et al,[38] 2015	1	1		1			1	1	1	1	0	0	0	0	0	1
Number of studies with feature present (of 35)	***24***	***35***		***26***			***24***	***26***	***34***	***30***	***2***	***3***	***3***	***7***	***4***	***35***

© 2022 Cook DA et al. *JAMA Network Open.*

Criteria (below) selected from the 1996 BMJ Guidelines;[40] see that publication for additional details on each item. 1 = present, 0 = absent; blank shaded cells indicate that this quality element was not relevant to this study or study classification (i.e., the outcome was not assessed).

- 8 The source(s) of effectiveness estimates used are stated.
- 9 Details of the design and results of effectiveness study are given (if based on a single study).
- 16 Quantities of resources are reported separately from their unit costs.
- 17 Methods for the estimation of quantities and unit costs are described.
- 18 Currency and price data are recorded.
- 22 Time horizon of costs and benefits is stated.
- 23 The discount rate(s) is stated.
- 24 The choice of rate(s) is justified.
- 25 An explanation is given if costs or benefits are not discounted.
- 27 The approach to sensitivity analysis is given.
- 30 Relevant alternatives are compared [operationally defined as comparing cost for 2 or more CPD approaches).
- 31 Incremental analysis is reported [operationally defined to include incremental cost-effectiveness ratio or net benefit].
- 32 Major outcomes are presented in a disaggregated as well as aggregated form.

© 2022 Cook DA et al. *JAMA Network Open.*

eFigure 2. Cost-effectiveness and cost-benefit (education costs and clinical outcomes): Dominance ranking matrix

Panel A. Comparison with alternate CPD approach

	Higher education cost	Same education cost	Lower education cost
**Favorable effect**	**5 studies (6 comparisons):**<br>• Soumerai et al,[2] 1986: Outreach vs mailed<br>• Lopez-Picazo et al,[31] 2011: Outreach: 1-on-1 vs group<br>• Qureshi et al,[32] 2011: Multimodal vs mailed<br>• Watson et al,[19] 2001: Outreach (1-on-1) + mailed vs mailed<br>• Simon et al,[25] 2005 (2 comparisons): Outreach: 1-on-1 vs group; Outreach (1-on-1) vs mailed	--	--
**Similar effect**	--	--	--
**Unfavorable effect**	--	--	--

Panel B. Comparison with no intervention

	Higher education cost	Same education cost	Lower education cost
**Favorable effect**	**9 studies (all compared CPD vs no intervention or baseline):**<br>• Soumerai et al,[2] 1986<br>• Lopez-Picazo et al,[31] 2011<br>• Qureshi et al,[32] 2011<br>• Siriwardena et al,[27] 2007<br>• Landgren et al,[3] 1988<br>• Zimmerman et al,[9] 1994<br>• McNulty et al,[17] 2000<br>• Watson et al,[19] 2001<br>• Butler et al,[34] 2012	--	--
**Similar effect**	--	--	--
**Unfavorable effect**	--	--	--

The Dominance Ranking Matrix organizes all studies according to the direction of effect for comparisons of costs (higher, same, or lower) and effectiveness (favorable, similar, unfavorable). Effectiveness was measured using clinical outcomes related to prescribing (prescription rates, errors, or costs).

© 2022 Cook DA et al. *JAMA Network Open.*

- Studies in the upper right (green cells) show dominance in favor of the intervention – lower or similar education cost combined with favorable or similar effectiveness; such interventions should be accepted over the alternative. (There were no such studies found in this review.)
- Studies in the lower left corner (red cells) show dominance against the intervention – higher or similar education cost combined with unfavorable or similar effectiveness; such interventions should be rejected in favor of the alternative. (There were no such studies found in this review.)
- Studies along the diagonal (orange cells) show unclear dominance – higher education cost and favorable effectiveness, lower cost and unfavorable effectiveness, or similar cost and effectiveness; judgment is thus required in deciding which intervention is preferable, considering factors such as incremental cost effectiveness measures and priorities/willingness to pay.

In this figure, all results refer to comparison of the first intervention relative to the second (i.e., the hypothesis that the first has favorable effectiveness / lower cost).

© 2022 Cook DA et al. *JAMA Network Open.*

References

1. Moleski RJ, Andriole VT. Role of the infectious disease specialist in containing costs of antibiotics in the hospital. *Rev Infect Dis*. 1986;8:488-493.
2. Soumerai SB, Avorn J. Economic and policy analysis of university-based drug "detailing". *Medical Care*. 1986;24:313-331.
3. Landgren FT, Harvey KJ, Mashford ML, Moulds RF, Guthrie B, Hemming M. Changing antibiotic prescribing by educational marketing. *Medical Journal of Australia*. 1988;149:595-599.
4. Raisch DW, Bootman JL, Larson LN, McGhan WF. Improving antiulcer agent prescribing in a health maintenance organization. *Am J Hosp Pharm*. 1990;47:1766-1773.
5. Friis H, Bro F, Mabeck CE, Vejlsgaard R. Changes in prescription of antibiotics in general practice in relation to different strategies for drug information. *Dan Med Bull*. 1991;38:380-382.
6. Stuart ME, Handley MA, Chamberlain MA, Wallach RW, Penna PM, Stergachis A. Successful implementation of a guideline program for the rational use of lipid-lowering drugs. *Hmo Pract*. 1991;5:198-204.
7. Hadbavny AM, Hoyt JW. Promotion of cost-effective benzodiazepine sedation. *Am J Hosp Pharm*. 1993;50:660-661.
8. Weir B. Antibiotic prophylaxis in cesarean section: use of cost per case comparison to influence prescribing practices. *Hospital Formulary*. 1993;28:285-286, 289-290.
9. Zimmerman DR, Collins TM, Lipowski EE, Sainfort F. Evaluation of a DUR intervention: a case study of histamine antagonists. *Inquiry*. 1994;31:89-101.
10. Ziskind AA, Portelli J, Rodriguez S, Stafford JL, Herzog WR, Knox JG, et al. Successful use of education and cost-based feedback strategies to reduce physician utilization of low-osmolality contrast agents in the cardiac catheterization laboratory. *American Journal of Cardiology*. 1994;73:1219-1221.
11. Bausch J. [The pharmacotherapy circle--a promising way for improving quality in primary medical care]. *Z Arztl Fortbild (Jena)*. 1995;89:406-414.
12. Schectman JM, Schroth WS, Elinsky EG, Ott JE. The effect of education and drug samples on antihistamine prescribing costs in an HMO. *Hmo Pract*. 1996;10:119-122.
13. von Ferber L, Bausch J, Schubert I, Koster I, Ihle P. [Drug therapy courses for family physicians--advanced education in pharmacotherapy]. *Zeitschrift fur Arztliche Fortbildung und Qualitatssicherung*. 1997;91:767-772.
14. Boreen D, Juge D, Stahl J, Torborg S. Effects of a physician education program on prescribing of HMG-CoA reductase inhibitors. *Formulary*. 1998;33:802-809.
15. Hux JE, Melady MP, DeBoer D. Confidential prescriber feedback and education to improve antibiotic use in primary care: a controlled trial. *Cmaj*. 1999;161:388-392.
16. Use physician education to cut prescription drug costs. *Capitation Management Report*. 1999;6:100-103, 197.
17. McNulty CA, Kane A, Foy CJ, Sykes J, Saunders P, Cartwright KA. Primary care workshops can reduce and rationalize antibiotic prescribing. *J Antimicrob Chemother*. 2000;46:493-499.
18. Valori RM, Brown CM, Strangeways P, Bradburn M. Reducing community dyspepsia drug costs: A controlled trial. *Gut*. 2001;49:495-501.
19. Watson M, Gunnell D, Peters T, Brookes S, Sharp D. Guidelines and educational outreach visits from community pharmacists to improve prescribing in general practice: a randomised controlled trial. *J Health Serv Res Policy*. 2001;6:207-213.
20. Bell N. Antibiotic resistance: the Iowa experience. *American Journal of Managed Care*. 2002;8:988-994.
21. Bernal-Delgado E, Galeote-Mayor M, Pradas-Arnal F, Peiro-Moreno S. Evidence based educational outreach visits: effects on prescriptions of non-steroidal anti-inflammatory drugs. *J Epidemiol Community Health*. 2002;56:653-658.
22. Dobscha SK, Anderson TA, Hoffman WF, Winterbottom LM, Turner EH, Snodgrass LS, et al. Strategies to decrease costs of prescribing selective serotonin reuptake inhibitors at a VA Medical Center. *Psychiatric Services*. 2003;54:195-200.

23. Lutters M, Harbarth S, Janssens J-P, Freudiger H, Herrmann F, Michel J-P, et al. Effect of a comprehensive, multidisciplinary, educational program on the use of antibiotics in a geriatric university hospital. *Journal of the American Geriatrics Society*. 2004;52:112-116.
24. Madridejos-Mora R, Amado-Guirado E, Perez-Rodriguez MT. Effectiveness of the combination of feedback and educational recommendations for improving drug prescription in general practice. *Medical Care*. 2004;42:643-648.
25. Simon SR, Majumdar SR, Prosser LA, Salem-Schatz S, Warner C, Kleinman K, et al. Group versus individual academic detailing to improve the use of antihypertensive medications in primary care: a cluster-randomized controlled trial. *American Journal of Medicine*. 2005;118:521-528.
26. Chazan B, Turjeman RBZ, Frost Y, Besharat B, Tabenkin H, Stainberg A, et al. Antibiotic consumption successfully reduced by a community intervention program. *Israel Medical Association Journal*. 2007;9:16-20.
27. Siriwardena AN, Fairchild P, Gibson S, Sach T, Dewey M. Investigation of the effect of a countywide protected learning time scheme on prescribing rates of ramipril: interrupted time series study. *Family Practice*. 2007;24:26-33.
28. Apisarnthanarak A, Yatrasert A, Mundy LM, Thammasat University Antimicrobial Stewardship T. Impact of education and an antifungal stewardship program for candidiasis at a Thai tertiary care center. *Infect Control Hosp Epidemiol*. 2010;31:722-727.
29. Niquille A, Ruggli M, Buchmann M, Jordan D, Bugnon O. The nine-year sustained cost-containment impact of Swiss pilot physicians-pharmacists quality circles. *Annals of Pharmacotherapy*. 2010;44:650-657.
30. del Arco A, Tortajada B, de la Torre J, Olalla J, Padra JL, Montiel N, et al. [Results of a counselling programme in antibiotic treatment in a secondary hospital]. *Rev Esp Quimioter*. 2011;24:96-98.
31. Lopez-Picazo JJ, Ruiz JC, Sanchez JF, Ariza A, Aguilera B. A randomized trial of the effectiveness and efficiency of interventions to reduce potential drug interactions in primary care. *Am J Med Qual*. 2011;26:145-153.
32. Qureshi NA, Neyaz Y, Khoja T, Magzoub MA, Haycox A, Walley T. Effectiveness of three interventions on primary care physicians' medication prescribing in Riyadh City, Saudi Arabia.[Erratum appears in East Mediterr Health J. 2011 Mar;17(3):249]. *Eastern Mediterranean Health Journal*. 2011;17:172-179.
33. Weiss K, Blais R, Fortin A, Lantin S, Gaudet M. Impact of a multipronged education strategy on antibiotic prescribing in Quebec, Canada. *Clinical Infectious Diseases*. 2011;53:433-439.
34. Butler CC, Simpson SA, Dunstan F, Rollnick S, Cohen D, Gillespie D, et al. Effectiveness of multifaceted educational programme to reduce antibiotic dispensing in primary care: practice based randomised controlled trial. *Bmj*. 2012;344:d8173.
35. Dormuth CR, Carney G, Taylor S, Bassett K, Maclure M. A randomized trial assessing the impact of a personal printed feedback portrait on statin prescribing in primary care. *Journal of Continuing Education in the Health Professions*. 2012;32:153-162.
36. Le Corvoisier P, Renard V, Roudot-Thoraval F, Cazalens T, Veerabudun K, Canoui-Poitrine F, et al. Long-term effects of an educational seminar on antibiotic prescribing by GPs: a randomised controlled trial. *British Journal of General Practice*. 2013;63:e455-464.
37. Holuby RS, Pellegrin KL, Barbato A, Ciarleglio A. Recruitment of rural healthcare professionals for live continuing education. *Med*. 2015;20:28958.
38. Pechlivanoglou P, Wieringa JE, de Jager T, Postma MJ. The effect of financial and educational incentives on rational prescribing. A state-space approach. *Health Econ*. 2015;24:439-453.
39. Cook DA, Reed DA. Appraising the quality of medical education research methods: the Medical Education Research Study Quality Instrument and the Newcastle-Ottawa Scale-Education. *Acad Med*. 2015;90:1067-1076.
40. Drummond MF, Jefferson TO. Guidelines for authors and peer reviewers of economic submissions to the BMJ. The BMJ Economic Evaluation Working Party. *BMJ*. 1996;313:275-283.

© 2022 Cook DA et al. *JAMA Network Open.*
